# Molecular glues that inhibit specific Zn^2+^-dependent DUB activity and inflammation

**DOI:** 10.1101/2024.09.07.611787

**Published:** 2024-09-07

**Authors:** Francesca Chandler, Poli Adi Narayana Reddy, Smita Bhutda, Rebecca L. Ross, Miriam Walden, Kieran Walker, Stefano Di Donato, Joel A. Cassel, Michael A. Prakesch, Ahmed Aman, Alessandro Datti, Lisa J. Campbell, Martina Foglizzo, Lillie Bell, Daniel N. Stein, James R. Ault, Rima S. Al-awar, Antonio N. Calabrese, Frank Sicheri, Francesco Del Galdo, Joseph M. Salvino, Roger A. Greenberg, Elton Zeqiraj

**Affiliations:** 1 Astbury Centre for Structural Molecular Biology, School of Molecular and Cellular Biology, Faculty of Biological Sciences, University of Leeds, Leeds, UK,; 2 The Wistar Cancer Center for Molecular Screening, The Wistar Institute, Philadelphia, PA, USA,; 3 Department of Cancer Biology, Penn Center for Genome Integrity, Basser Center for BRCA, Perelman School of Medicine, University of Pennsylvania, Philadelphia, PA, USA,; 4 Leeds Institute of Rheumatic and Musculoskeletal Medicine, Faculty of Medicine and Health, University of Leeds, Leeds, UK,; 5 NIHR Leeds Biomedical Research Centre, Leeds Teaching Hospitals, NHS Trust, Chapel Allerton Hospital, Leeds, UK; 6 Drug Discovery Program, Ontario Institute for Cancer Research, Toronto, ON, Canada,; 7 Department of Agriculture, Food, and Environmental Sciences, University of Perugia, Perugia, Italy,; 8 Department of Pharmacology and Toxicology, University of Toronto, Toronto, ON, Canada,; 9 Centre for Systems Biology, Lunenfeld-Tanenbaum Research Institute, Sinai Health System, Toronto, ON, Canada,; 10 Department of Molecular Genetics, University of Toronto, Toronto, ON, Canada,; 11 Department of Biochemistry, University of Toronto, Toronto, ON, Canada

## Abstract

Deubiquitylases (DUBs) play a pivotal role in cell signalling and are often regulated by homo- or hetero-interactions within protein complexes. The BRCC36 isopeptidase complex (BRISC) regulates inflammatory signalling by selectively cleaving K63-linked polyubiquitin chains on Type I interferon receptors (IFNAR1). BRCC36 is a Zn^2+^-dependent JAMM/MPN DUB, a challenging ubiquitin protease class for the design of selective inhibitors. We identified first-in-class DUB inhibitors that act as BRISC molecular glues (BLUEs). BLUEs inhibit DUB activity by stabilising a BRISC dimer consisting of 16 subunits. The BLUE-stabilised BRISC dimer is an autoinhibited conformation, whereby the active sites and interactions with the recruiting subunit SHMT2 are blocked. This unique mode of action leads to highly selective inhibitors for BRISC over related complexes with the same catalytic subunit, splice variants and other JAMM/MPN DUBs. Structure-guided inhibitor resistant mutants confirm BLUEs on-target activity in cells, and BLUE treatment results in reduced interferon-stimulated gene (ISG) expression in human peripheral blood mononuclear cells from Scleroderma patients, a disease linked with aberrant IFNAR1 activation. BLUEs represent a new class of molecules with potential utility in Type I interferon-mediated diseases and a template for designing selective inhibitors of large protein complexes by promoting protein-protein interactions instead of blocking them.

## Introduction

There are over 100 human DUBs, which control cellular signalling by dictating the activity, localisation, or stability of protein substrates^1–5^. DUB dysfunction is implicated in a range of pathologies, including autoimmune disorders, cancers, metabolic diseases, and neurodegeneration^6–8^. Consequently, DUBs remain attractive therapeutic targets and are the focus of many drug discovery efforts^9,10^.

BRCC36 is a JAMM (JAB1, MOV34, and MPR1, Pad1 N-terminal (MPN)) metalloenzyme DUB family and selectively cleaves lysine 63-linked ubiquitin (K63-Ub) chains^11,12^. BRCC36 is present in two distinct macromolecular assemblies: a cytoplasmic BRCC36 isopeptidase complex (BRISC), and the nuclear Abraxas1 isopeptidase complex (ARISC). The BRISC complex regulates Type I interferon signalling by deubiquitylating and stabilising Type I interferon (IFNAR1) receptors, whilst the ARISC complex interacts with the tumour suppressor protein BRCA1 and localises to double-stranded DNA breaks to facilitate DNA damage repair^13–15^. BRCC36 (MPN^+^) requires a pseudo-DUB partner for enzymatic activity. This occurs through a stable heterodimeric complex with either Abraxas1 (MPN^−^) in the nucleus, or Abraxas2 (MPN^−^) in the cytoplasm^12,16–18^. BRISC and ARISC complexes contain two additional proteins, BRCC45 and MERIT40, and form dimer of hetero-tetramer assemblies with a 2:2:2:2 stoichiometry^16–19^. These eight subunit enzyme complexes require additional interacting partners for cellular function. BRISC forms a complex with a metabolic enzyme, serine hydroxymethyltransferase 2 (SHMT2) for targeting to IFNAR1 receptors, and loss of this interaction leads to a reduction in interferon signalling^20^. ARISC forms the BRCA1-A complex with BRCA1, BARD1, and RAP80 in the nucleus to facilitate recruitment to DNA double strand breaks^13–15^. Recent cryo-electron microscopy structures of the BRISC-SHMT2 complex revealed a U-shaped assembly of the BRISC complex, whereby the BRCC36-Abraxas2 heterodimer bridges two BRCC45-MERIT40 “arms”^20,21^. A similar overall architecture was also observed for ARISC structures^19,21,22^.

BRISC-mediated deubiquitylation of IFNAR1 receptors promotes JAK/STAT signalling and expression of interferon (IFN) stimulated genes (ISGs)^23^. Elevated ISG expression is associated with autoimmune diseases, including Systemic Lupus Erythematosus (SLE)^24^, Rheumatoid Arthritis (RA)^25^, and Systemic Sclerosis (SSc)^26^. BRISC-deficient mice are protected from elevated interferon signalling and certain forms of inflammation^23^. Therefore, targeting BRISC with small molecule inhibitors represents a therapeutic strategy to reduce persistent inflammation and subsequent autoimmune disease driven pathology.

Significant progress has been made in selectively targeting the ubiquitin specific protease (USP) family DUBs^27–31^. These hold promise as potential therapeutics and as tool compounds to understand DUB biology. However, most inhibitors of the JAMM/MPN family of DUBs are broad-spectrum zinc chelators and there are currently no selective inhibitors for BRCC36 complexes^11,32^. Capzimin, a quinoline-8-thiol (8TQ) derivative targets the active site zinc of proteasomal subunit Rpn11, but also inhibits BRCC36 and AMSH^33^. Inhibitors of the JAMM domain containing de-neddylase, CSN5, also engage the catalytic zinc, but show specificity for CSN5 over AMSH and PSMD14^34^. Thus, whilst major progress has been made in DUB inhibitor development^35^, small-molecule inhibitors of the JAMM/MPN DUBs exclusively target the conserved zinc binding pocket, which makes the development of selective inhibitors challenging.

Molecular glues (MGs) are defined as small molecule stabilisers of protein-protein interactions^36,37^. Such compounds act as immunosuppressants (e.g. cyclosporin A^38,39^, rapamycin) and natural degraders (e.g. auxin in plants^37^). Immune-modulatory imide drugs (IMiDs), such as thalidomide, are MGs which induce protein degradation by stabilising an interaction between the E3 ligase cereblon and neo-substrates^40^. As such, MGs are an attractive class of compounds for regulating protein stability and degradation, however, MGs for DUBs have not been reported.

We describe first-in-class, selective BRISC inhibitors and define a unique mechanism of action for a DUB inhibitor. Cryo-EM structures of BRISC in complex with small molecule inhibitors reveal the molecular basis for selectivity and compound mechanism of action. The BRISC inhibitors identified here act as molecular glues and do not engage the active site zinc. Instead, BRISC molecular glues (BLUEs) inhibit DUB activity by stabilising a BRISC conformer that occludes the BRCC36 active site from accepting ubiquitin chains for cleavage. We show target engagement in cells through structure-guided mutagenesis and cell-based studies, and we further validate inhibitor mechanism of action on human cells following IFN stimulation and from patients with aberrant IFNAR1 activation. Overall, this study showcases the therapeutic potential of MG compounds which promote specific protein-protein interactions to achieve selective inhibition of macromolecular complexes.

## Results

### Identification of first-in-class, selective BRISC inhibitors

**Figure 1.**
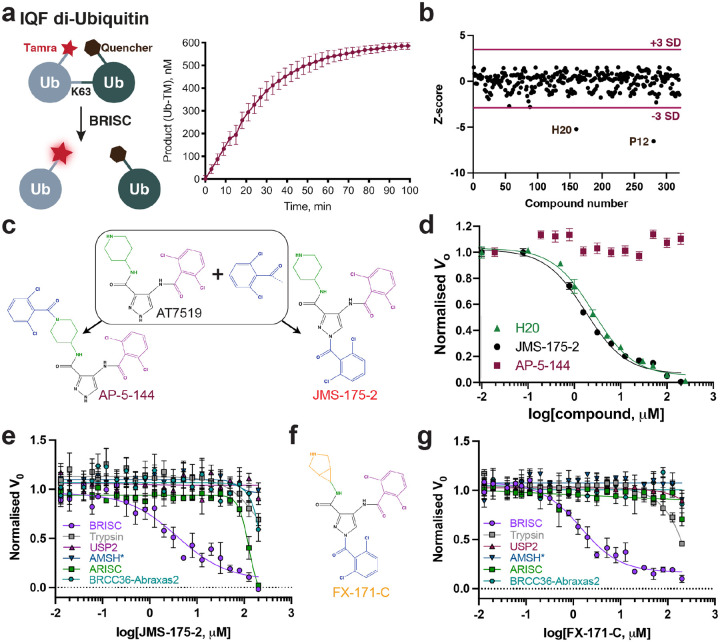
Fluorescence-based screen to identify first-in-class JAMM inhibitors **a,** Schematic of a TAMRA-linked internally-quenched fluorescent (IQF) di-ubiquitin substrate (left) and reaction progress curve of BRISC DUB activity (right). **b,** Z-score normalisation of 320 compounds from an in-house kinase-directed inhibitor library and identification of hit compounds in wells H20 and P12. SD = standard deviation. **c,** Chemical structures of AT7519 and of two isomers with an additional 2,6-dichlorobenzaldehyde moiety. **d,** Dose-response inhibition of BRISC activity by the H20 compound and the two potential isomers, AP-5–144 and JMS-175–2. **e, g,** Dose-response inhibition of trypsin, USP2 and JAMM/MPN DUB enzymes AMSH* (a STAM2-AMSH fusion^44^), BRISC, ARISC, and BRCC36-Abraxas2 by the indicated compounds. Data points in **d, e,** and **g,** are mean ± SEM of three independent experiments carried out in duplicate. **f,** Chemical structure of the FX-171-C compound.

We designed a biochemical screen to identify small-molecule inhibitors of BRISC by measuring activity using a K63-linked di-ubiquitin substrate with an internally-quenched fluorophore (Fig. 1a). Increased fluorescence was detected after DUB cleavage over time, enabling continuous readout of DUB activity (Fig. 1a). The di-ubiquitin cleavage assay was used to screen an in-house compound library of 320 published and custom-made kinase inhibitors. Two hits were identified as compounds AT7519 (well H20) and YM201636 (well P12) (Fig. 1b). The selectivity of these compounds was assessed against the broad-spectrum ubiquitin specific peptidase 2 (USP2), and the serine protease trypsin, which cleave K63-Ub substrate under the same assay conditions. YM201636 (well P12) inhibited BRISC, trypsin, and USP2 suggesting this is a non-specific inhibitor, whilst what we presumed to be compound AT7519 (well H20) showed selective inhibition of BRISC DUB activity **(Extended Data Fig. 1a)**. To further validate the hit compound in well H20, we purchased AT7519 from two commercial vendors, Synkinase and Selleckchem. Curiously, neither inhibited BRISC DUB activity in the IQF assay **(Extended Data Fig. 1b)**. UV-Vis spectroscopy analyses showed a different pattern for the compound in well H20 compared to the purchased AT7519 compounds **(Extended Data Fig. 1c)**, suggesting the compound in well H20 was different to AT7519. Liquid chromatography-mass spectrometry (LC-MS) revealed the H20 compound was pure, with a mass of 555.55 Da instead of the expected mass of 382.25 Da^41^ **(Extended Data Fig. 1d)**. This mass difference is consistent with the addition of a 2,6-dichlorobenzaldehyde group, which we reasoned could have been inadvertently added during chemical synthesis at either the piperidine or pyrazole ring. We synthesised both possible isomers: AP-5–144 and JMS-175–2 (Fig. 1c), and tested their inhibitory effects against BRISC. We found JMS-175–2 matched the profile of compound in well H20, inhibiting BRISC with an IC_50_ of 3.8 μM (Fig. 1d). Consistent with the JMS-175–2 structure, mass spectrometry fragmentation analyses showed that compound H20 contains the 2,6-dichlorobenzaldehyde modification at the pyrazole ring, and not the piperidine ring **(Extended Data Fig. 1e)**. The AP-5–144 isomer did not inhibit BRISC DUB activity and using fragmentation analyses we confirmed AP-5–144 did not match the chemical structure of compound H20 **(Extended Data Fig. 1e)**. These data confirm the chemical structure of the compound in well H20 and led to the serendipitous identification of the BRISC inhibitor JMS-175–2.

We next determined the selectivity of inhibitors for the BRISC DUB beyond USP2 and trypsin. AMSH is a related JAMM/MPN DUB which, like BRCC36, selectively cleaves K63-linked polyubiquitin chains^42,43^. JMS-175–2 did not inhibit AMSH* (a STAM2-AMSH fusion)^44^ (Fig. 1e), showing it is selective for BRISC over other zinc-dependent DUBs. Remarkably, JMS-175–2 did not inhibit the nuclear ARISC complex, which shares three of the four BRISC subunits, including the catalytic subunit BRCC36 (Fig. 1e). A related analogue, FX-171-C (Fig. 1f), had a moderately improved IC_50_ of 1.4 μM compared to JMS-175–2 (IC_50_ = 3.8 μM), and also retained selectivity for BRISC against other JAMM/MPN DUBs (Fig. 1g). These data confirm JMS-175–2 series are highly selective BRISC inhibitors and suggest the specificity is conferred, in part, by the Abraxas2 subunit which is substituted for Abraxas1 in the ARISC complex. Curiously, JMS-175–2 and FX-171-C did not inhibit the minimally active BRCC36-Abraxas2 complex, which indicates that the “arm” regions containing BRCC45 and MERIT40 also contribute to the inhibitor selectivity profile (Fig. 1e).

Interestingly, we noticed a biphasic mode of inhibition **(Extended Data Fig. 2a)**, and enzyme activity inhibition plots at different substrate concentrations suggested JMS-175–2 and FX-171-C act as noncompetitive inhibitors **(Extended Data Fig. 2b)**. The strong selectivity of the JMS-175–2 and FX-171-C compounds and a noncompetitive mode of inhibition indicate these inhibitors do not target the Zn^2+^ active site, unlike previously described JAMM/MPN inhibitors^32–34^.

We used Western blotting to demonstrate both JMS-175–2 and FX-171-C inhibit BRISC-mediated cleavage of polyubiquitin chains **(Extended Data Fig. 2c)**. The other possible JMS-175–2 stereoisomer, AP-5–144, did not inhibit BRISC cleavage of tetraubiquitin chains, consistent with the di-ubiquitin fluorescence assay (Fig. 1d). We also observed dose-dependent inhibition of tetra-ubiquitin chain cleavage with FX-171-C **(Extended Data Fig. 2d)**. These experiments identify the first selective inhibitors of BRISC, and allude to a unique mechanism of action whereby the Abraxas2 pseudo-DUB subunit, and the BRCC45-MERIT40 “arms” contribute to selective inhibition of the cytoplasmic BRISC complex.

### BRISC inhibitors stabilise an autoinhibited dimer conformation

**Figure 2.**
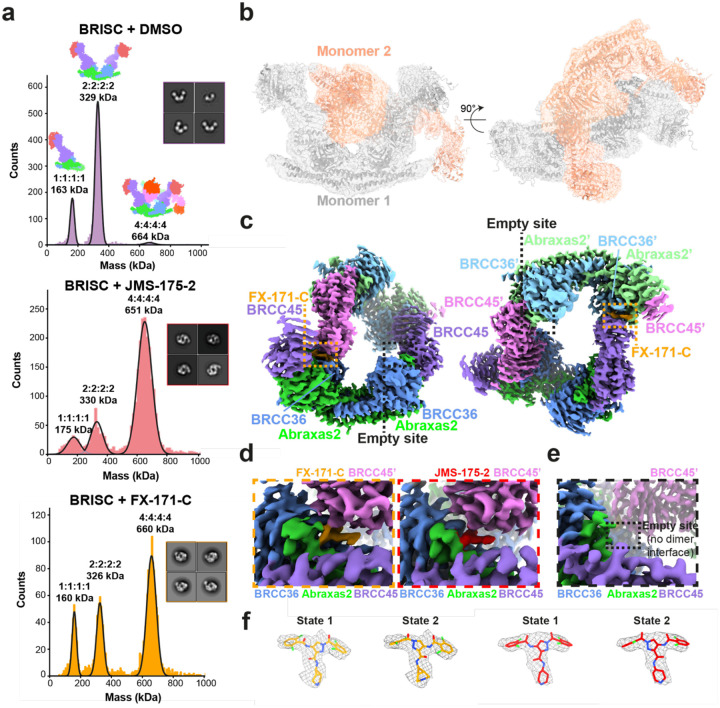
Inhibitors stabilise an inhibited BRISC dimer **a,** Mass photometry histograms of purified BRISC in absence (DMSO, top) and presence of inhibitors (JMS-175–2, middle, FX-171-C, bottom), and corresponding negative stain EM 2D classes of BRISC mixed with DMSO or inhibitors (insets). **b,** Cryo-EM density of BRISC-FX-171-C co-structure at 3.0 Å. BRISC monomers are shown as grey and salmon cartoon models, and fitted to the cryo-EM map shown as a transparent surface at 0.00224 threshold. The C-termini of BRCC45 (reisdues 275–383) and MERIT40 are rigid-body fitted into the density. **c,** BRISC-FX-171-C cryo-EM density map at 0.0165 threshold. BRISC subunits are coloured by chain. The density corresponding to FX-171-C is coloured orange and highlighted in orange boxes. **d,** Close-up views of the indicated inhibitor density comparing FX-171-C (left) and JMS-175–2 (right) binding sites. **e,** Cryo-EM density at the equivalent sites of BRCC36, Abraxas2, and BRCC45’ in the BRISC-FX-171-C co-structure where there is no dimer interface, and no additional density corresponding to FX-171-C. **f,** Structures of FX-171-C and JMS-175–2 modelled in State 1 and State 2. Cryo-EM density after focused refinement represented as a mesh.

To understand the molecular basis of BRISC inhibition by the new inhibitor series, and to determine the small molecule binding site, we characterised the complex by mass photometry and cryo-electron microscopy (cryo-EM). Single molecule mass photometry measurements in the absence of any inhibitors revealed three populations of purified BRISC complexes. The major population corresponded to a single BRISC complex with four subunits at a 2:2:2:2 ratio (Fig. 2a, **top**), consistent with negative stain EM 2D class averages (Fig. 2a, **top**) and with previous studies^17,19,20^. We also observed a population at 163 kDa which may correspond to a dissociated 1:1:1:1 complex, or the BRCC36-Abraxas2 super dimer and minimally active complex^18^. Surprisingly, we also observed a third population, consisting of 2–5% of the particles, with an estimated molecular weight of 664 kDa. This corresponds to the mass of two BRISC “monomer” complexes with a predicted 4:4:4:4 stoichiometry.

Consistent with these measurements, we observed a higher molecular weight BRISC species in cryo-EM data. We observed BRISC “dimer” complexes in 2D class averages containing ~5% of the particles **(Extended Data Fig. 3a)**. The majority of particles correspond to a monomeric complex **(Extended Data Figs. 3b, 3c, Extended Data Table 1)**, but a low resolution cryo-EM reconstruction of these particles indicated density for two BRISC monomers **(Extended Data Fig. 3d, Extended Data Table 1)**. The conformation of this dimeric BRISC species was different from the symmetric BRISC and ARISC dimers previously reported in glutaraldehyde cross-linked samples imaged by negative stain EM^19,21^ **(Extended Data Fig. 3e)**. This suggests that BRISC has a propensity to dimerise, raising the possibility that these low-level dimers may be regulated or stabilised by ligand binding.

Interestingly, incubating purified BRISC with JMS-175–2 and FX-171-C resulted in a considerable mass shift to the 4:4:4:4 complex, which suggests the inhibitor promotes BRISC dimer formation (Fig. 2a). Negative stain electron microscopy confirmed the oligomeric state on inhibitor addition, with 2D class averages that look like two U-shaped BRISC assemblies (Fig. 2a, inserts). Using native mass spectrometry we confirmed the inhibitor-induced mass corresponds to a dimeric BRISC complex and BRISC dimers with 4:4:4:4 stoichiometry were detected after addition of JMS-175–2 and FX-171-C compounds **(Extended Data Figs. 3f, 3g)**. Importantly, we also observed a dose-dependent increase in dimer formation by mass photometry for both JMS-175–2 and FX-171-C **(Extended Data Fig. 3h)**. These data suggest an unexpected mode of action where inhibitor binding promotes a stable BRISC dimer complex of 16 subunits and molecular weight of 655 kDa.

### Cryo-EM structures reveal BRISC inhibitors act as molecular glues

To determine the precise mechanism by which a small molecule can induce formation of a multimeric DUB complex, we solved co-structures of BRISC complexes, bound to FX-171-C and JMS-175–2. We observed a high proportion (>95%) of BRISC dimers after preincubation with each inhibitor and after 3D refinement and postprocessing, we obtained cryo-EM maps at 3.0 Å (FX-171-C) and 3.3 Å resolution (JMS-175–2) (Fig. 2b, **Extended Data Figs. 4a–f, Extended Data Table 1**). The BRISC-inhibitor structures consist of a BRISC dimer, with density for all 16 subunits (stoichiometry 4:4:4:4), where the BRCC45-MERIT40 “arms” of one BRISC monomer hooks around the BRCC45-MERIT40 arm of a neighbouring BRISC molecule (BRISC’), bridging the BRCC36-Abraxas2 super dimer (Fig. 2b). Modelling of K63-linked di-ubiquitin substrate in this conformation suggests that the recruitment of a second BRISC octamer occludes the BRCC36 active sites by sterically blocking chain binding and catalysis **(Extended Data Fig. 4g)**.

We observe the highest resolution (2.8–3.6 Å) in the core of the BRISC dimer structures, consisting of the BRCC36-Abraxas2 super dimer and the BRCC45’ subunit which forms the dimer interface (Fig. 2c, **Extended Data Figs. 4c, 4f**). The resolution is lower (7–12 Å) for the extreme C-termini of BRCC45 and MERIT40 (arm regions), therefore limiting accurate model building of these regions. This is due to the flexible nature of the arm regions and is consistent with our previous observations of the BRISC-SHMT2 cryo-EM structure^20^. Due to the lower resolution of the map beyond the second ubiquitin E2 variant (UEV) domain of BRCC45 and for MERIT40, we rigid body fitted BRCC45 UEV-C (residues 275–383) and MERIT40 from previous BRISC-SHMT2 structures^20,21^.

The binding interface formed by BRCC36, Abraxas2, and BRCC45’ is also formed at the opposite site of the dimer structure (BRCC36’, Abraxas2’, BRCC45). At both interfaces, we observe additional density which is not attributed to either BRISC monomer and the density has the size and shape expected for each inhibitor (Fig. 2d). Importantly, the equivalent BRCC36-Abraxas2 surface that is not in contact with BRCC45’ from an opposing BRISC monomer does not contain this extra cryo-EM density (Fig. 2e). The extra density is present in the same location for both the FX-171-C and JMS-175–2 maps (Fig. 2d), indicating a similar mode of binding for both compounds. Due to the slight tilting of the BRISC’ monomer resulting in an asymmetric dimer, there is one inhibitor bound per BRISC molecule, and two inhibitors per BRISC dimer (4:4:4:4:2 stoichiometry).

Focused refinement using a mask comprising the core of the BRISC dimer moderately improved the density for the FX-171-C compound **(Extended Data Figs. 4h, 4i)**. Likewise, applying a mask on the highest resolution half of the JMS-175–2 map also improved the density for JMS-175–2 **(Extended Data Figs. 4j, 4k)**. Due to the presence of two dichlorobenzene rings in each compound, we were unable to unambiguously determine the orientation of the dichlorobenzene moieties in the cryo-EM densities and have modelled the ligands in two orientations: State 1 and State 2 (Fig. 2f).

Next, we examined the conformational changes induced by FX-171-C using differential HDX-MS analysis. Measuring differences in deuterium uptake, detected at the peptide level, in the absence and presence of FX-171-C enabled us to analyse the structural rearrangement after inhibitor binding **(Extended Data Fig. 5a)**. For example, regions of protection upon FX-171-C addition were identified in BRCC36 (residues 111–135) and BRCC45 (residues 122–134), which are consistent with the small molecule binding site and interaction interfaces identified in our cryo-EM structures **(Extended Data Figs. 5b, 5c)**. We also observed deprotection of a BRCC36 peptide (142–149), indicative of a change in solvent accessibility near the enzyme active site.

Protected peptides in BRCC45 (residues 206–221, 311–327) suggest further interactions between BRCC45 subunits from opposing BRISC monomers **(Extended Data Fig. 5b)**. Moreover, deprotected peptides in the BRCC36-Abraxas2 coiled-coil and the C-termini of BRCC45 and MERIT40 subunits indicate additional and far-reaching conformational changes induced by inhibitor binding **(Extended Data Fig. 5b)**.

Collectively, these structural analyses establish the inhibitors are BRISC molecular glues (BLUEs) which stabilise two BRISC octamers to form a BRISC dimer with 16-subunits. BLUEs bind at a composite site of three interacting proteins: BRCC36 and Abraxas2 from one BRISC monomer and BRCC45’ from a second BRISC monomer. The inhibitor-induced dimer is an inactive conformation, whereby ubiquitin chain binding and processing is blocked.

### The BLUE binding pocket

The BLUE compound binding pocket is in close proximity to, but does not engage, the catalytic zinc and does not interact with BRCC36 active site residues **(Extended Data Fig. 5d)**, consistent with substrate kinetic analyses suggesting that BLUEs are non-competitive inhibitors **(Extended Data Figs. 2a, 2b)**. BLUE compounds are the first examples of non-competitive JAMM/MPN DUB inhibitors, as all known JAMM/MPN DUB inhibitors described to date target the zinc binding site^32–34,45^ **(Extended Data Fig. 5e)**. In addition to exploring a new binding site for JAMM/MPN DUBs, BLUE compound engagement of the middle BRCC45 ubiquitin E2 variant (UEV) domain highlights another unexpected compound binding surface in E2 folds. Unlike BAY 11–7082 and NSC697923, inhibitors of E2 Ubc13, BLUE compounds do not engage the UEV pseudo-catalytic site^46^, nor bind to an allosteric site exemplified by the Cdc34 E2 inhibitor, CC0651^47^ **(Extended Data Fig. 5f)**.

**Figure 3.**
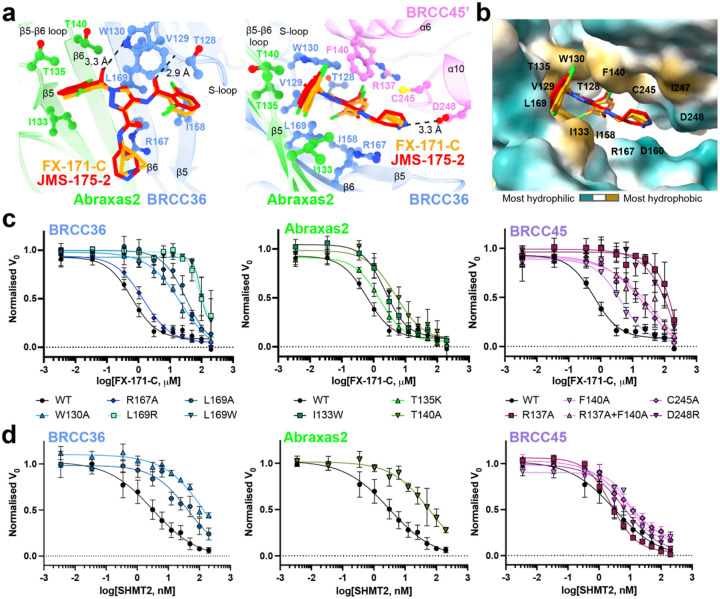
Analysis of the BLUE compound binding site **a,** Model of the FX-171-C and JMS-175–2 binding pocket at the interface of BRCC36, Abraxas2 and BRCC45. Hydrogen bonds represented by blue dashed lines and residues studied by mutagenesis indicated. **b,** The BLUE compound binding pocket shown as a surface and coloured by hydrophobicity. **c,** FX-171-C inhibition of BRISC DUB activity with BRCC36, Abraxas2 and BRCC45 mutants. **d**, SHMT2 inhibition of the same BRISC mutants as in **c,**. Data in **c,** and **d,** are mean ± SEM of three independent experiments carried out in duplicate.

The local resolution of our cryo-EM structures at the dimer interface is ~2.8 Å (FX-171-C map, (Fig. 2d)) and sufficient to identify residues from each BRISC monomer which contribute to the inhibitor binding pocket. In BRCC36, BLUEs bind between the S-loop (β4-α3) and the β5, β6-strands. In Abraxas2, BLUE compounds interact with the β5-strand and β5-β6 loop (Fig. 3a, **left**). Two α-helices (BRCC45 α6 and α10) from the BRCC45’ subunit also line the inhibitor binding pocket (Fig. 3a, **right**). The two dichlorobenzene moieties of JMS-175–2 and FX-171-C sit in a hydrophobic groove formed by BRCC36 S-loop residues T128 and W130, and residues I158 and L169 (Fig. 3b). Abraxas2 I133 and BRCC45’ F140, C245, and I247 also contribute to the hydrophobic binding pocket. The JMS-175–2 piperidine ring and FX-171-C pyrrolidine ring extend into a hydrophilic region encompassing BRCC36 D160 and R167, and BRCC45’ D248.

BRCC36 forms two hydrogen bonds with the BLUE compounds. In both State 1 and 2, the amide backbone of V129 and the W130 side-chain (BRCC36 S-loop) form hydrogen bonds with the two amide oxygens either side of the central pyrazole ring. BRCC45’ F140 forms aromatic stacking interactions with the central pyrazole ring, and the BRCC45’ D248 forms a hydrogen bond with the amine group in the JMS-175–2 piperidine or FX-171-C pyrrolidine ring (Fig. 3a). Consistent with this interaction, analogues containing methyl subsitutions of the piperidine ring showed reduced inhibition of BRISC activity **(Extended Data Figs. 6a, 6b)**. BRCC45’ R137 forms a hydrogen bond with the BRCC45’ loop containing C245 to stabilise the BRCC45’ α10 helix that lines the compound binding site.

### A human-specific BRCC36 loop promotes BRISC dimer formation

BLUE compounds are highly selective for BRISC over other JAMM/MPN DUBs, including the closely related ARISC complex which shares the BRCC36 catalytic subunit (Figs. 1e, 1g). Cryo-EM structures revealed BLUEs directly engage the Abraxas2 subunit. Sequence alignment of Abraxas1 (ARISC) and Abraxas2 (BRISC) illustrates divergence in the primary amino acid sequence near the BLUE compound binding site (β5–β6 loop) **(Extended Data Fig. 6c)**, which likely contributes to the selectivity of BLUEs for BRISC over ARISC.

To further probe compound selectivity, we tested FX-171-C inhibition of BRISC complexes from metazoan orthologues: mouse (*Mus musculus)*, zebrafish (*Danio rerio)*, and ant (*Camponotus floridanus)*. FX-171-C has the highest potency towards human BRISC over mouse and zebrafish BRISC, whilst there is no inhibition of ant BRISC **(Extended Data Fig. 6d)**. Analysing the BRISC-BLUE interaction interface explains the high specificity of BLUE compounds for human BRISC over ant BRISC. BRCC36 W130 and L169 and the Abraxas2 β5-β6 strands, which line the inhibitor binding pocket (Fig. 3a), are not conserved in *Cf*BRCC36 and *Cf*Abraxas2 **(Extended Data Figs. 6c, 6e)**. BRCC45 C245, which contributes to compound binding, is also not conserved in *Cf*BRISC **(Extended Data Fig. 6f)**.

Analysing the selectivity between human and mouse BRISC suggested a possible contributer of dimer formation. Human and mouse BRISC share over 97% sequence identity, yet FX-171-C is approximately ten times more potent as an inhibitor of human BRISC. The major difference between the two species is an extended loop region of 25 amino acids in human BRCC36 (residues 184–208) **(Extended Data Fig. 6e)**. Deletion of this loop in human BRISC reduced inhibitor sensitivity by approximately 10 fold, with a similar IC_50_ for mouse BRISC which lacks the same loop **(Extended Data Fig. 6d)**. Consistent with the idea that the loop region mediates dimer formation we observed fewer dimers for the human BRISCΔLoop construct upon inhibitor addition in mass photometry and negative stain EM **(Extended Data Figs. 6g, 6h)**. The cryo-EM density which corresponds to the BRCC36 loop extends towards an Abraxas2 subunit from the opposing BRISC molecule, further supporting the role for this loop in mediating BRISC dimerisation **(Extended Data Fig. 6i)**. Interestingly, humans have two BRCC36 isoforms with one lacking this loop region, suggesting that it is possible to design molecular glue compounds that display not only selectivity within the same enzyme family, but also across orthologous species and splice variants.

### BRISC-inhibitor interacting residues are important for inhibitor sensitivity *in vitro*

The cryo-EM structures of BRISC in complex with molecular glues allowed us to make selective mutations to probe the BRISC-BLUE interaction site and to assess the contribution of each interacting residue for inhibition. We mutated residues from BRCC36, Abraxas2, and BRCC45, and purified 15 mutant BRISC complexes from insect cells **(Extended Data Fig. 7a)**. Due to the close proximity of some residues to the BRCC36 active site, we assessed BRISC DUB activity against a fluorogenic di-ubiquitin substrate. Two BRCC36 mutants (T128P and I158K) were inactive, and we determined which mutations conferred a reduction in inhibitor sensitivity for the remaining 13 active mutant complexes **(Extended Data Fig. 7b)**.

BRCC36 W130A and L169A/R/W mutants showed severely reduced inhibition by FX-171-C (>100 fold over WT complex), whilst BRCC36 R167A remained inhibitor sensitive (Fig. 3c, **Extended Data Fig. 7c**). Abraxas2 mutant T140A was moderately affected, exhibiting an IC_50_ 10-fold higher than BRISC WT, while Abraxas2 I133W and T135K had moderate to little effect on inhibitor sensitivity (Fig. 3c, **Extended Data Fig. 7c**). We also mutated BRCC45’ residues (R137A, F140A, C245A, D248R) and all had reduced sensitivity to inhibition when compared with WT BRISC complexes (Fig. 3c, **Extended Data Fig. 7c**). These data validate the BLUE compound binding sites identified by cryo-EM, and the reduced sensitivity observed for the BRCC45’ mutants confirms the molecular gluing mechanism of inhibition.

### Molecular glues and SHMT2 share a binding pocket

The metabolic enzyme SHMT2 interacts with BRISC to regulate IFNAR1 signalling^20^. Interestingly, the SHMT2 binding site on BRISC overlaps with the BLUE compound binding site **(Extended Data Fig. 7d)**. Indeed, some of the residues we mutated to validate inhibitor binding also contribute to the SHMT2 interaction interface. As SHMT2 is a potent endogenous inhibitor of BRISC DUB activity^20^, we assessed if BRISC mutants were still inhibited by SHMT2 (Fig. 3d). BRCC36 W130A and L169A and Abraxas2 T140A show reduced BRISC inhibition by SHMT2, indicating these mutations also disrupt SHMT2 binding to BRISC. By contrast, the BRCC45 mutants were inhibited by SHMT2 with a similar IC_50_ to BRISC WT, which is consistent with these BRCC45 residues being far away from the BRISC-SHMT2 binding interface in the context of the BRISC monomer **(Extended Data Fig. 7e)**. Therefore, both the BLUE compounds and the endogenous inhibitor SHMT2 share a common interacting site, suggesting that one potential role for SHMT2 may be to prevent the formation of a BRISC dimer.

### BLUE compounds reduce interferon signalling

To probe the on-target effect of BLUE compounds in cells we generated BRCC45 KO MCF10A cells using CRISPR-*Cas*9-mediated genomic deletion **(Extended Data Fig. 8a)** and complemented BRCC45 KO with either Flag-BRCC45 WT or Flag-BRCC45 R137A **(Extended Data Fig. 8b)**. BRCC45 R137A mutation reduces FX-171-C inhibition by ~2 orders of magnitude without affecting SHMT2 binding (Figs. 3c, 3d). We confirmed by co-immunoprecipitation that the interactions with BRISC subunits BRCC36 and MERIT40 were maintained in the BRCC45 WT and BRCC45 R137A cell lines **(Extended Data Fig. 8c)**.

Cells were challenged with IFNα to stimulate IFNAR1 signalling and ISG expression. An increase in STAT1 phosphorylation was observed for all cell lines, and less STAT1 phosphorylation was observed in BRCC45 KO cells **(Extended Data Fig. 8d)**. Following IFNα stimulation, we used quantitative PCR with reverse transcription (qRT-PCR) to measure changes in gene expression for five ISGs: ISG15, IFIT1, IFIT2, IFITM1, and CXCL10. The differences in gene expression between sgROSA (control sgRNA), WT and R137A cell lines were non-significant, with the exception of ISG15, which had higher gene expression in BRCC45 WT cells, but still comparable to BRCC45 R137A **(Extended Data Fig. 8e)**.

To measure the effect of BLUE compounds on ISG expression, we compared BRCC45 WT and BRCC45 R137A cell lines with JMS-175–2 and FX-171-C. For additional controls, we tested two further compounds: FX-171-A and AP-5–145. FX-171-A has a similar chemical structure to FX-171-C, but is less potent (IC_50_ = 6.8 μM) **(Extended Data Figs. 8f, 8g)**. AP-5–145 is an N-methylated analogue of AP-5–144, the other possible stereoisomer synthesised at the beginning of this study and has no inhibitory effect against BRISC (Fig. 1c, **Extended Data Fig. 8f**). We added a a methyl group to the pyrazole ring to avoid potential CDK2 inhibition in cells and to increase cell permeability when used as a negative control.

**Figure 4.**
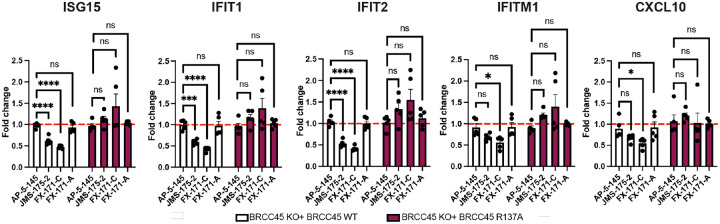
BLUE compounds reduce interferon-stimulated gene (ISG) expression in cells MCF10A Cas9 cells expressing BRCC45 wild-type (WT) and BRCC45 R137A were treated with/without hIFNα (75 ng/mL) and either 2.5 μM inhibitor (JMS-175–2, FX-171-C, FX-171-A), negative control AP-5–145 or DMSO (0.1 %) for 4 hours. Expression of indicated interferon-induced genes normalised to 18s rRNA are presented as fold change to own IFN + DMSO treated control (n=4). One-way ANOVA was used to compare statistical significance between AP-5–145 vs. JMS-175–2, AP-5–145 vs. FX-171-C, and AP-5–145 vs. FX-171-A. Error bars represent ± SEM.

Treatment of BRCC45 WT cells with 2.5 μM JMS-175–2 and FX-171-C reduced gene expression for all five ISGs (Fig. 4). Critically, a reduction in ISG expression was not observed in cells harbouring the BRCC45 R137A mutation, which reduces BLUE inhibition of BRISC, indicating on-target BLUE activity in cells.

**Figure 5.**
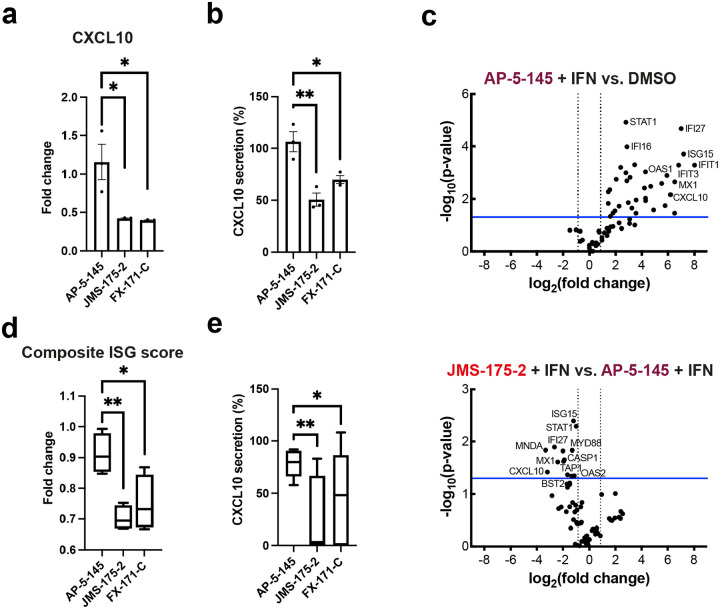
BLUE compounds reduce interferon-stimulated gene (ISG) expression in healthy and SSc PBMCs **a,** Healthy PBMCs treated with/without IFN (20 ng/mL) and with either BRISC inhibitors (2 μM JMS-175–2 and FX-171-C), AP-5–145 negative control or DMSO control (0.1%) for 16 h. CXCL10 gene expression analysis was normalised to GAPDH for n=3 donors, and expressed as fold change to own IFN+DMSO control. Paired two-tailed student t test was used to compare between AP-5–145 vs. JMS-175–2 and AP-5–145 vs. FX-171-C for statistical significance. Error bars represent ± SEM. **b,** PBMCs supernatant from **a** was used to measure CXCL10 secretion and expressed as percentage to own IFN + DMSO control (100%). Paired two-tailed student t test was used to compare between AP-5–145 vs. JMS-175–2 and AP-5–145 vs. FX-171-C for statistical significance. Error bars represent ± SEM. **c,** Type I IFN signalling gene expression analysis (n=67 normalised for housekeeping genes; ACTB, GAPDH, HPRT1, RPLP0) of healthy control PBMCs stimulated with IFN. Volcano plot illustrating genes increased with addition of AP-5–145 + IFN compared to DMSO only (n=3) (top), or addition of JMS-175–2 + IFN compared to AP-5–145 + IFN (n=3) (bottom). **d,** SSc PBMCs (n=20 donors) were treated with DMSO, AP-5–145, FX-171-C, or JMS-175–2 for 16 h. Composite IFN score (CXCL10, IFIT1, ISG15, MX1) gene expression analysis between conditions relative to each donors DMSO control are illustrated as box plot of median and interquartile ranges. Paired two-tailed student t test was used to compare AP-5–145 vs. JMS-175–2 and FX-171-C. **e,** SSc PBMCs supernatant CXCL10 secretion is expressed as percentage to own DMSO control, and illustrated as a box plot of median and interquartile ranges (n=7 donors). Paired two-tailed student t test was used to compare AP-5–145 vs. JMS-175–2 and FX-171-C.

We also studied the effects of BLUE compound treatment in peripheral blood mononuclear cells (PBMCs) from healthy volunteers upon IFNα stimulation. Consistent with our analyses in MCF10A cells, we observed a reduction in CXCL10 expression for PBMCs treated with JMS-175–2 and FX-171-C, but not AP-5–145 (Fig. 5a). We also observed less CXCL10 protein secretion in BLUE-treated PBMCs, measured by ELISA (Fig. 5b). For a more comprehensive analysis of how BLUE compounds modulate the IFN gene signature, we performed qRT-PCR to analyse the expression of 67 ISGs, including ISG15, IFIT1, IFIT2, IFITM1, CXCL10 and MX1. 35 ISGs were significantly upregulated by IFN, with no difference in ISG expression profile between DMSO or AP-5–145 (Fig. 5c, **Extended Data Fig. 9a**). Treatment with JMS-175–2 suppressed the IFN-induced signature compared to AP-5–145 (Fig. 5c), with significant reduction in 14 of the 35 ISGs observed in AP-5–145 **(Extended Data Fig. 9c)**. Interestingly, for FX-171-C-treated PBMCs, there was less reduction in ISG expression compared to AP-5–145 control, which indicates the BLUE compounds may be metabolised differently, at least in PBMCs **(Extended Data Figs. 9b, 9c)**.

To assess the activity of BLUE compounds in the context of autoimmune disease associated with aberrant Type I IFN activation, we measured ISG levels in PBMCs from 20 patients affected by Scleroderma (SSc)^26^, using qRT-PCR. We used a composite ISG score, including CXCL10, IFIT1, ISG15 and MX1 relative to GAPDH^48^. The expression of these genes are increased by recombinant IFN in healthy PBMC (Fig. 5c, **Extended Data Fig. 9a**). Treatment of SSc PBMCs with JMS-175–2 or FX-171-C reduced the composite ISG score relative to AP-5–145 (Fig. 5d, **Extended Data Figs. 9d, 9e**). We also observed a reduction in CXCL10 protein secretion following treatment of PBMCs with JMS-175–2 or FX-171-C (Fig. 5e, **Extended Data Fig. 9f**). For a more comprehensive analysis of BLUE compound modulation of ISG expression in SSc PBMCs we selected three responders and performed qRT-PCR to monitor the expression of the 35 ISGs induced in healthy PBMCs with recombinant IFN **(Extended Data Fig. 9g)**. Both JMS-175–2 and FX-171-C statistically reduced expression of these ISGs relative to AP-5–145 **(Extended Fig. 9g)**.

## Discussion

Deubiquitylating enzymes regulate many aspects of cellular signalling and are attractive drug targets due to their diverse roles in cancer, neurodegeneration, inflammation, and immunity^10,49–51^. The BRISC DUB complex regulates Type I interferon signalling and thus is a promising target for the treatment of autoimmune diseases^24–26^. However, the high conservation among active sites of JAMM/MPN DUBs makes the development of selective BRISC inhibitors challenging. Using a combination of fluorescence-based screening and biochemical assays we identified first-in-class, selective inhibitors of the cytoplasmic BRISC DUB complex. The screening platform and orthogonal validation assays developed here serve as a proof-of-concept study that can be readily adapted for systematic and high-throughput screening efforts of BRISC and other DUBs.

**Figure 6.**
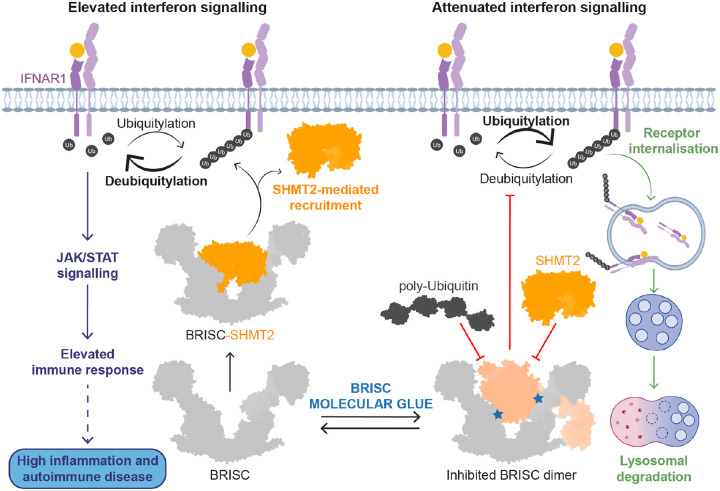
Proposed model of BLUE compound mode of action Interferon binding to IFNAR1 receptors triggers JAK/STAT signalling and an elevated immune response. Interferon also initiates IFNAR1 receptor ubiquitylation (K63-linked), receptor internalisation and lysosomal degradation. The BRISC-SHMT2 complex is required for deubiquitylation of IFNAR1. BRISC is recruited to IFNAR1/2 through interactions with SHMT2 to promote sustained interferon signalling and inflammation. BLUE compounds (blue stars) promote formation of a BRISC dimer complex, which sterically hinders SHMT2 and poly-Ub binding).

BRISC inhibitors are molecular glues, which induce the formation of an inhibited BRISC dimer. We showed target engagement in cells using an inhibitor resistant mutation, BRCC45 R137A. In addition, we measured a reduction in interferon-stimulated gene expression in MCF10A cells and PBMCs from healthy and scleroderma patients after treatment with BLUE compounds. Based on current and published reports about IFNAR1 being a substrate of BRISC, we propose a model where reduced BRISC activity results in hyper-ubiquitylation of IFNAR1 and accelerated receptor degradation, leading to reduced inflammatory signalling (Figure 6). Combining structural analysis of current models of BRISC interactions with SHMT2 and ubiquitin, we propose that BLUEs stabilise a new autoinhibited BRISC dimer.

DUBs are regulated through autoinhibited conformations, protein-protein interactions, and interactions with pseudo-DUB partners. To our knowledge this is the first example of a DUB molecular glue. This concept could be applied to other DUBs or macromolecular complexes to develop selective inhibitors by promoting interactions instead of using the classical approach of breaking protein-ligand or protein-protein interactions. Molecular glues which stabilise autoinhibited conformations offer a unique opportunity to exploit naturally existing mechanisms and achieve selectivity for DUBs with highly conserved active sites and which also exist as dimers (e.g. USP25 and USP28 (ref.^52^). Thus far, only cross-reactive inhibitors of the USP25 and USP28 DUBs have been described^53,54^. Therefore, molecular glues that stabilise the autoinhibited forms offer a route to achieving selective inhibition and development of effective therapeutics^55^.

Indeed, there are examples of USP inhibitors which block catalysis by stabilising an inhibited conformation and misaligning active site residues, e.g. USP7 inhibitors FT671 and FT827^28^, or the cross-reactive USP11/15 inhibitor mitoxantrone^56,57^. Unlike these examples, BLUE compounds stabilise an inhibited conformation without disrupting the BRCC36 catalytic site **(Extended Data Fig. 5d)**. This is also the first example of a new class of JAMM/MPN DUB inhibitor, as all other JAMM/MPN inhibitors, including the selective CSN5 inhibitors^34^, interact with the catalytic zinc **(Extended Data Fig. 5e)**.

BLUE compounds achieve exquisite selectivity by exploiting a binding site at the interface of three different proteins (Fig. 2d). Most of the currently identified molecular glues contact two different proteins, with a few exceptions. Natural compounds Cyclosporin A (CsA) and FK506 were the first characterised molecular glues and exert immunosuppressive activity by disrupting signalling events mediated by calcineurin^38^. CsA and FK506 stabilise interactions between three different proteins. Both compounds contact calcineurin A and calcineurin B. FK506 induces an interaction with FKBP12 and CsA induces an interaction with cyclophilin^38,58,59^. Interestingly, in the case of the BLUE compounds, they interact at the interface of a DUB, a pseudo-DUB and a UEV domain, contacting a previously unexplored UEV site.

DUB complexes use protein-protein interactions to regulate enzymatic activity, cellular localisation, and substrate binding. The subcellular localisation, function and activity of BRCC36 is regulated by the interaction of pseudo-DUBs, namely Abraxas1 and Abraxas2^16^. Here, we show additional regulation of BRISC activity through dimerisation. The identification of BRISC dimers in the absence of small molecules alludes to a mechanism of action whereby the molecular glues stabilise a low affinity interaction between BRISC molecules. Indeed, the ability of molecular glues to enhance the affinity between proteins which already have a pre-existing low affinity interaction is what distinguishes them from bifunctional molecules such as PROTACs^60^. The asymmetric dimer conformation blocks the BRCC36 active site, predicted polyubiquitin binding sites **(Extended Data Fig. 4g)**, and the BLUE binding pocket overlaps with the binding interface of the endogenous inhibitor SHMT2 **(Extended Data Fig. 7d)**. Therefore, it is possible that the asymmetric dimer represents an autoinhibited BRISC conformation which is sampled by a small population of the complex in the absence of external stimuli (i.e. small molecule binding or as yet, unidentified mechanisms). Determining the functional importance of the different BRISC dimers and identifying their potential regulation is an important area of future exploration.

Testing BLUE inhibition of BRISC orthologues highlighted a human-specific BRCC36 loop which is important for inhibitor sensitivity and dimer formation. Moreover, a human splice isoform lacks this loop region (residues 184–208). This difference between mouse and human forms calls for a cautious approach if murine models are used for efficacy or translational studies. Moreover, in cryo-EM maps of the BRISC dimer, the flexible BRCC36 loop extends towards the opposite BRISC molecule, and acts to stabilise the BRISC dimer **(Extended Data Fig. 6i)**. These insights suggest the autoinhibited conformation is prevalent for the human BRISC complex and driven by the human-specific BRCC36 loop, although further investigation is required to elucidate the regulation and formation of BRISC dimers in cells.

## Materials and methods

### Reagents

Reagents and antibodies were purchased from the following commercial sources:

Anti-Flag M2 Affinity Gel beads (Sigma-Aldrich, #A2220), Recombinant Human IFN-α2 (carrier free) (Biolegend, #592702) were purchased. The following antibodies were used for immunoblotting: Anti-BRCC45 (Abcam, #ab177960), Anti-GAPDH (Cell Signaling Technology, #2118S), Anti-Merit40 (Cell Signaling Technology, #12711S), Anti-BRCC36 (Abcam, #ab108411), Anti-Phospho-Stat1 (Tyr701) (Cell Signaling Technology, #9167S), Anti-α Actin (Santa Cruz Biotechnology, SC-#32251), HRP-conjugated anti-Ubiquitin (P4D1) (Santa Cruz Biotechnology, SC-#8017). POWER RT-qPCR reagents including SYBR Green PCR Master mix (#4367659) and cDNA Reverse Transcription Kit (#4368814) were purchased from Allied Biosystems. RNeasy mini kit (#74104) and RNase free DNase (#79254) were purchased from Qiagen. The 4–12% Bis-Tris SDS-PAGE gels, cell culture media, horse serum and other materials for tissue culture were purchased from Invitrogen. LipoD293 (SigmaGen, #SL100668) was used for all the transfections to generate knock out cell lines. Media supplements including human insulin solution (Santa Cruz Biotechnology, #sc-360248), cholera toxin from vibrio (Sigma Aldrich, #C8052–2MG), recombinant human EGF (PeproTech, #AF-100-15-100ug), hydrocortisone (Sigma-Aldrich, #H-0888) were used to grow MCF10A cells.

### Expression and purification of DUB complexes

Four-subunit human BRISC and ARISC complexes (full-length (FL) BRISC, ARISC (FL), BRISCΔNΔC(MERIT40ΔN, Abraxas2ΔC), BRCC36-Abraxas2, BRISCΔLoop, *Dr*BRISCΔNΔC, *Cf*BRISCΔNΔC) were cloned using the MultiBac system and co-expressed in *Spodoptera frugiperda* (*Sf*9) insect cells^61^. BRISC mutants and *Mm*BRISC were cloned into pFastBac vectors in the Bac-to-Bac system (ThermoFisher), baculoviruses were generated in *Sf*9 cells and used for co-infection of *Trichoplusia ni* (*Tni*) cells. All BRISC complexes were purified as previously described^18,20^.

USP2 with an N-terminal His-tag was purchased from Addgene (plasmid #36894). AMSH*, an AMSH-STAM fusion^44^, with an N-terminal His-tag was purchased from Addgene (pOPINB-AMSH*, plasmid #66712). His-USP2 and His-AMSH* were expressed in *Escherichia coli* BL21 (DE3) cells. Cells were grown at 37 °C in Terrific Broth (TB) medium, induced with 0.5 mM isopropyl-β-D-1-thiogalactopyranoside (IPTG) and grown overnight at 18 °C. For purification, cell pellets were resuspended in lysis buffer containing 50 mM Tris-HCl pH 7.6, 300 mM NaCl, 20 mM imidazole, 5% glycerol, 0.075% β -mercaptoethanol, 1 mM benzamidine, 0.8 mM phenylmethylsulfonyl fluoride (PMSF) and 0.3 mg/mL lysozyme. Cells were lysed by sonication (1 s on, 1 s off for a total of 16 minutes) and cleared by centrifugation at 18,000 g. The clarified lysate was incubated with Ni-NTA beads (Cytiva) for 1 hour at 4 °C, before washing with wash buffer containing 50 mM Tris-HCl pH 7.6, 300 mM NaCl, 20 mM imidazole, 5% glycerol, 0.075% β -mercaptoethanol, and 1 mM benzamidine and a high salt buffer containing 500 mM NaCl. The protein was eluted with elution buffer containing 50 mM Tris-HCl pH 7.6, 300 mM NaCl, 120 mM imidazole, 5% glycerol, 0.075% β-mercaptoethanol, and 1 mM benzamidine. The elutions containing His-USP2 or His-AMSH* were dialysed overnight with thrombin (His-USP2) or 3C PreScisssion protease (His-AMSH*). After dialysis, cleaved samples were incubated with Ni-NTA beads and washed with wash buffer. The cleaved fractions were concentrated and loaded onto a Superdex 75 10/300 column (Cytiva) equilibrated with 25 mM HEPES pH 7.5, 150 mM NaCl, and 1 mM TCEP.

### Compound library screening

BRISC DUB activity was measured at room temperature (RT) using 1 nM BRISC and 500 nM internally quenched flurescence (IQF) diUb K63 substrate (Lifesensors, K63–3) in the presence of DMSO and compounds at 10 μM (final concentrations). Assay was performed in a 384-well black flat-bottom low-flange plate (Corning; 35373) in buffer containing 50 mM HEPES-NaOH pH 7.0, 100 mM NaCl, 1 mg/mL BSA, 1 mM DTT, and 0.03% v/v Brij-35. Ten microlitres of x2 concentrated enzyme stock was dispensed followed by transfer of 200 nl of compounds (1 mM stock) using a 384-pin tool and a 15 min incubation at room temperature. Ten microlitres of x2 concentrated substrate stock was added and the reaction was monitored by measuring fluorescence intensity (excitation, 540 nm; emission, 580 nm) after 20 min incubation at RT and ~50% of substrate was consumed. Orthogonal assays for verification of H20 and P12 hit compounds using USP2 (100 nM) and Trypsin (125 nM) were performed in identical conditions.

### LCMS and MS-MS analysis

LC MS-MS analysis was carried out in a UPLC BEH C18 (2.1 × 50 mm, 1.7 μm) column using ACQUITY UPLC II system. The mobile phase was 0.1% formic acid in water (solvent A) and 0.1% formic acid in acetonitrile (solvent B). A gradient starting at 95% solvent A going to 5% in 4.5 minutes, holding for 0.5 minutes, going back to 95% in 0.5 minutes and equilibrating the column for 1 minute was employed. A Waters Synapt G2S QTof mass spectrometer equipped with an electrospray ionization source was used for mass spectrometric analysis. MassLynx (v4.1) was used for data analysis. The MS parameters for LCMS analysis were frequency of 15 s, cone voltage of 25 V, capillary voltage was 3 kV. For MS-MS spectra we used: MS-MS range of m/z 50–900, scan time of 0.1 s, collision energy ramp of 30–60 volts. Compound identifications were performed using accurate mass analysis and MS-MS fragmentation analysis. Absorbance spectra was measured using a UV-VIS nanodrop spectrophotometer N8000 (Thermofisher).

### Synthesis of BRISC inhibitors

Reaction schemes for the synthesis of JMS-175–2 and FX-171-C are outlined in **Extended Data Fig. 10**. All commercially obtained solvents and reagents were used as received. Flash column chromatography was performed using silica gel 60 (230–400 mesh). Analytical thin layer chromatography (TLC) was carried out on Merck silica gel plates with QF-254 indicator and visualized by UV, PMA, or KMnO4. 1H NMR spectra were recorded on a Bruker Advance 400. Chemical shifts are reported in parts per million (ppm, δ) using the residual solvent line as a reference. Splitting patterns are designated using the following abbreviations: s, singlet; d, doublet; t, triplet; dd, doublet of doublet; m, multiplet; br, broad. Coupling constants (J) are reported in hertz (Hz). Tetramethylsilane was used as an internal standard for proton nuclear magnetic resonance for samples run in CDCl3 or DMSO-d6. LC−MS data were acquired on a Waters Acquity UPLC/MS system equipped with a UPLC binary pump, an SQD 3100 mass spectrometer with an electrospray ionization (ESI) source and a PDA detector (210−400 nm). High-resolution mass spectra were obtained using the Q Exactive HF-X mass spectrometer which provided high resolution, accurate mass, and total ion and extracted ion chromatograms. All compounds tested were present within a 5 ppm mass error. The purity of all final compounds was determined by HPLC, and the compounds are at least ≥95% pure. 1H NMR spectra, mass spectra and HPLC traces are shown in Supplementary Information.

#### Tert-butyl 6-(4-nitro-1H-pyrazole-3-carboxamido)-3-azabicyclo[3.1.0]hexane-3-carboxylate (3; AP-09–123).

To a stirred solution of 4-nitro-1H-pyrazole-3-carboxylic acid (1.0 g; 6.36 mmol) **(1)** and tert-butyl 6-amino-3-azabicyclo[3.1.0]hexane-3-carboxylate (1.325 g; 6.68 mmol) **(2)** in 20 mL of dry CH_2_Cl_2_ at 0 °C was added diisopropyl ethyl amine (5.56 mL; 31.82 mmol) and T3P 50% solution in CH_2_Cl_2_ by weight (5.26 g; 8.28 mmol) simultaneously dropwise. The reaction mixture was stirred for an additional 1 h at 0 °C. Completion of the reaction was confirmed by LC−MS. The product was extracted with CH_2_Cl_2_ (10 mL × 2) and washed with 1N *aq* HCl (20 mL), saturated *aq* NaHCO_3_ (20 mL), brine solution (20 mL) and dried over anhydrous Na_2_SO_4_. The solvent was evaporated under reduced pressure to yield the crude product, which was purified by flash column chromatography to afford the title compound (**3**) as white solid (1.74 g; 5.16 mmol, 82%) which was confirmed by^1^ H NMR and MS. ^1^H NMR (400 MHz, DMSO) δ 8.91 – 8.64 (m, 2H), 3.66 – 3.43 (m, 4H), 2.48–2.40 (m, 1H), 1.75 (s, 2H), 1.39 (d, *J* = 7.4 Hz, 9H). Mass m/z: calculated for C_14_H_20_N_5_O_5_^+^ [M + H] ^+^, 338.15; found, 338.56.

#### Tert-butyl 6-(4-amino-1H-pyrazole-3-carboxamido)-3-azabicyclo[3.1.0]hexane-3-carboxylate (4; AP-09–124).

At room temperature, a suspension of tert-butyl 4-(4-nitro-1H-pyrazole-3-carboxamido)piperidine-1-carboxylate (1.7 g; 5.04 mmol) **(3)** and Pd/C (170 mg) in DMF (10 mL) and EtOH (30 mL) was stirred for 2 h under H_2_ at room temperature. Completion of the reaction was confirmed by TLC, the reaction mixture was filtered using Celite, the filtrate was concentrated under reduced pressure to obtain a residue, which was diluted with ethyl acetate and washed with cold brine solution and then dried over anhydrous Na_2_SO_4_. The solvent was evaporated under reduced pressure to yield the crude product, which was purified by flash column chromatography to afford the title compound (**4**) as a light brown solid (1.28 g; 4.18 mmol, (83%) which was confirmed by ^1^H NMR and MS. ^1^H NMR (400 MHz, DMSO) δ 12.54 (s, 1H), 7.98 (d, *J* = 4.0 Hz, 1H), 7.07 (d, *J* = 16.3 Hz, 1H), 4.57 (s, 2H), 3.49 (d, *J* = 10.8 Hz, 2H), 3.40–3.18 (m, 2H), 2.42 – 2.30 (m, 1H), 1.75 (d, *J* = 22.5 Hz, 2H), 1.39 (s, 9H). Mass m/z: calculated for C_14_H_21_N_5_NaO_3_^+^ [M + Na] ^+^, 330.15; found, 330.11.

#### Tert-butyl 6-(4-(2,6-dichlorobenzamido)-1-(2,6-dichlorobenzoyl)-1H-pyrazole-3-carboxamido)-3-azabicyclo[3.1.0]hexane-3-carboxylate (5; AP-09–125).

To a stirred solution of tert-butyl 6-(4-amino-1H-pyrazole-3-carboxamido)-3-azabicyclo[3.1.0]hexane-3-carboxylate (178 mg; 0.58 mmol) **(4)** in 5 mL of dry CH_2_Cl_2_ at 0 °C was added triethyl amine (0.81 mL; 5.79 mmol) and 2,6-dichlorobenzoyl chloride (727 mg; 3.47 mmol) simultaneously dropwise. The reaction mixture was slowly brought to room temperature and stirred for 16 h. Completion of the reaction was confirmed by LC−MS. The reaction mixture was quenched with 10 mL of cold water. The product was extracted with CH_2_Cl_2_ and washed with 1N *aq* HCl (10 mL), saturated *aq* NaHCO_3_ (10 mL), brine solution (10 mL) and dried over anhydrous Na_2_SO_4_. The solvent was evaporated under reduced pressure to yield the crude product, which was purified by flash column chromatography to afford the title compound (**5**) as a white solid (151 mg; 0.23 mmol, 40%) which was confirmed by ^1^H NMR and MS. ^1^H NMR (400 MHz, DMSO) δ 10.66 (s, 1H), 9.03 (s, 1H), 8.75 (s, 1H), 7.89–7.62 (m, 3H), 7.61–7.29 (m, 2H), 4.14 – 3.91 (m, 2H), 3.45 (d, *J* = 9.8 Hz, 2H), 2.35 (s, 1H), 1.81 (s, 2H), 1.47 – 1.26 (m, 9H). Mass m/z: calculated for C_28_H_25_Cl_4_N_5_NaO_5_^+^ [M + Na] ^+^, 674.05; found, 673.99.

#### N-(3-azabicyclo[3.1.0]hexan-6-yl)-4-(2,6-dichlorobenzamido)-1-(2,6-dichlorobenzoyl)-1H-pyrazole-3-carboxamide (6; FX-171-C (AP-09–131)).

To a stirred solution of tert-butyl 6-(4-(2,6-dichlorobenzamido)-1-(2,6-dichlorobenzoyl)-1H-pyrazole-3-carboxamido)-3-azabicyclo[3.1.0]hexane-3-carboxylate (**5**) (60 mg; 0.09 mmol) in 2 mL CH_2_Cl_2_ at 0 °C was added 0.5 mL of TFA. The reaction mixture was then warmed to room temperature and stirred for 3 h. Completion of the reaction was confirmed by LC−MS. Volatiles were evaporated under reduced pressure to yield the crude product was purified by flash column chromatography to afford the title compound (**6**) as white solid (51 mg; 0.08 mmol, 85 %) as confirmed by ^1^H NMR and MS. ^1^H NMR (400 MHz, MeOD) δ 9.23 (s, 1H), 7.59 – 7.45 (m, 3H), 7.45 – 7.36 (m, 3H), 3.67 – 3.39 (m, 4H), 2.71 (s, 1H), 2.08 (s, 2H). Mass m/z: calculated for C_28_H_26_Cl_4_N_5_O_5_^+^ [M + Na] ^+^, 552.07; found, 552.22.

#### Tert-butyl 4-(4-nitro-1H-pyrazole-3-carboxamido)piperidine-1-carboxylate (8; AP-09–090B).

To a stirred solution of 4-nitro-1H-pyrazole-3-carboxylic acid (1.0 g; 6.36 mmol) **(8)** and tert-butyl 4-aminopiperidine-1-carboxylate (1.401 g; 7.0 mmol) **(2)** in 20 mL of dry CH_2_Cl_2_ at 0 °C were added diisopropyl ethyl amine (4.43 mL; 25.46 mmol) and T3P 50% solution in CH_2_Cl_2_ by weight (4.858 g; 7.64 mmol) simultaneously dropwise.

The reaction mixture was stirred for an additional 1 h at 0 °C. Completion of the reaction was confirmed by LC−MS. The product was extracted with CH_2_Cl_2_ (10 mL × 2) and washed with 1N *aq* HCl (20 mL), saturated *aq* NaHCO_3_ (20 mL), brine solution (20 mL) and dried over anhydrous Na_2_SO_4_. The solvent was evaporated under reduced pressure to yield the crude product, which was purified by flash column chromatography to afford the title compound (**8**) as white solid (1.685 g; 4.97 mmol, 78 %). The product was confirmed by ^1^H NMR and MS. ^1^H NMR (400 MHz, DMSO) δ 14.15 (s, 1H), 8.69 (d, J = 59.8 Hz, 2H), 4.17 – 3.68 (m, 4H), 2.92 (s, 3H), 1.88 – 1.70 (m, 2H), 1.51 – 1.22 (m, 9H). Mass m/z: calculated for C_14_H_21_N_5_NaO_5_^+^ [M + Na] ^+^, 362.14; found, 362.15.

#### Tert-butyl 4-(4-amino-1H-pyrazole-3-carboxamido)piperidine-1-carboxylate (9; AP-09–091).

At room temperature, a suspension of tert-butyl 4-(4-nitro-1H-pyrazole-3-carboxamido)piperidine-1-carboxylate (**8**) (1.6 g, 2.27 mmol) and Pd/C (160 mg) in DMF (10 mL) and EtOH (30 mL) was stirred for 2 h under H_2_ at room temperature. Completion of the reaction was confirmed by TLC, the reaction mixture was filtered using Celite, the filtrate was concentrated under reduced pressure to obtain the crude residue which was diluted in ethyl acetate and washed with cold brine solution and then dried over anhydrous Na_2_SO_4_. The solvent was evaporated under reduced pressure to yield the crude product, which was purified by flash column chromatography to afford the title compound (**9**) as a light brown solid (1.241 g, 4.01 mmol, 85%). The product was confirmed by ^1^H NMR and MS. ^1^H NMR (400 MHz, CDCl_3_) δ 10.57 – 9.86 (m, 1H), 7.13 (s, 1H), 6.83 (s, 1H), 4.22 – 3.86 (m, 4H), 3.49 (s, 1H), 2.93 (dd, *J* = 21.1, 8.6 Hz, 2H), 1.98 (dd, *J* = 12.5, 2.8 Hz, 2H), 1.46 (s, 9H). Mass m/z: calculated for C_14_H_23_N_5_NaO_3_^+^ [M + Na] ^+^, 332.17; found, 332.11.

#### Tert-butyl 4-(4-(2,6-dichlorobenzamido)-1-(2,6-dichlorobenzoyl)-1H-pyrazole-3-carboxamido) piperidine −1-carboxylate (10; AP-09–120B).

To a stirred solution of tert-butyl 4-(4-amino-1H-pyrazole-3-carboxamido)piperidine-1-carboxylate (**9**) (150 mg; 0.48 mmol) in 5 mL of dry CH_2_Cl_2_ at 0 °C was added triethyl amine (0.67 mL; 4.85 mmol) and 2,6-dichlorobenzoyl chloride (727 mg; 2.91 mmol) simultaneously dropwise. The reaction mixture was slowly brought to room temperature and stirred for 16 h. Completion of the reaction was confirmed by LC−MS. The reaction mixture was quenched with 10 mL of cold water. The product was extracted with CH_2_Cl_2_ and washed with 1N *aq* HCl (10 mL), saturated *aq* NaHCO_3_ (10 mL), brine solution (10 mL) and dried over anhydrous Na_2_SO_4_. The solvent was evaporated under reduced pressure to yield the crude product, which was purified by flash column chromatography to afford the title compound (**10**) as a white solid (133 mg; 0.20 mmol, 42%). The product was confirmed by ^1^H NMR and MS. ^1^H NMR (400 MHz, DMSO) δ 10.66 (s, 1H), 9.03 (s, 1H), 8.50 (s, 1H), 7.70 (s, 3H), 7.64 – 7.45 (m, 3H), 3.90 (s, 3H), 2.74 (s, 2H), 1.66 (t, J = 19.4 Hz, 2H), 1.55 – 1.31 (s, 9H). Mass m/z: calculated for C_28_H_27_Cl_4_N_5_NaO_5_^+^ [M + Na] ^+^, 676.09; found, 676.0.

#### 4-(2,6-dichlorobenzamido)-1-(2,6-dichlorobenzoyl)-N-(piperidin-4-yl)-1H-pyrazole-3-carboxamide (11; JMS-175–2 (AP-09–126)).

To a stirred solution of tert-butyl 4-(4-(2,6-dichlorobenzamido)-1-(2,6-dichlorobenzoyl)-1H-pyrazole-3-carboxamido)piperidine-1-carboxylate (**10**) (100 mg; 0.152 mmol) in 4 mL CH_2_Cl_2_ at 0 °C was added 1 mL of TFA. The reaction mixture was then warmed to room temperature and stirred for 3 h. Completion of the reaction was confirmed by LC− MS. Volatiles were evaporated under reduced pressure to yield the crude product which was purified by flash column chromatography to afford the title compound (11) as a white solid (76 mg; 0.14 mmol, 90%) which was confirmed by 1H NMR and MS. ^1^H NMR (400 MHz, CDCl_3_) δ 9.79 (s, 1H), 9.26 (s, 2H), 7.56–7.40 (m, 3H), 7.39–7.25 (m, 3H), 6.64 (s, 1H), 4.20–4.02(m, 1H), 3.57–3.32 (m, 2H), 3.04–2.81 (m, 2H), 2.26–2.07 m, 2H), 1.95–1.73 (m, 2H). Mass m/z: calculated for C_23_H_20_Cl_4_N_5_O_3_^+^ [M + Na] ^+^, 554.03; found, 554.02.

### DUB activity assays (IQF)

IQF assays were performed in DUB reaction buffer containing 50 mM HEPES-NaOH pH 7.0, 100 mM NaCl, 0.1 mg/mL BSA, 1 mM DTT, and 0.03% v/v Brij-35. To assess inhibitor potency, inhibitors were diluted in DMSO up to a final concentration of 200 μM and incubated with the target enzyme for 15 minutes at room temperature. The final concentration of BRISC WT, all BRISC mutants, *Hs*BRISCDLoop, *Mm*BRISC and *Dr*BRISCDNDC was 1 nM. Other enzymes were tested at the following concentrations: 5 nM ARISC, 10 nM *Cf*BRISCDNDC, 250 nM AMSH* (a STAM2-AMSH fusion^44^), 500 nM USP2, and 1 μM trypsin (Sigma, 9002-07-7).

Michaelis-Menten analysis in the absence and prescence of increasing concentrations of either JMS-175–2 or FX-171-C were performed in DUB reaction buffer. BRISC concentration was 1 nM and IQF K63-linked di-ubiquitin substrate was used from 0 to 1 μM. Michaelis-Menten analyses and Lineweaver-Burk plots were calculated using GraphPad Prism (v9.0).

To assess enzymatic activity, BRISC complexes were diluted to concentrations between 1 nM and 50 nM. For SHMT2 assays, SHMT2(A285T) was diluted in 20 mM MES (pH 6.5), 500 mM NaCl and 2 mM TCEP up to a final concentration of 200 nM. SHMT2 was incubated with enzyme for 15 minutes at room temperature prior to addition of substrate. 20 μL enzyme reactions were carried out in 384-well black flat-bottom low flange plates (Corning; 35373). DUB activity was measured using internally quenched fluorescent (IQF) K63-linked diUb (Lifesensors, catalogue number: DU6303) at 50 nM. Cleaved di-ubiquitin was monitored by measuring fluorescence intensity (Ex. 544 nm, Em. 575 nm; dichroic mirror 560 nm). Fluorescence intensity was measured every minute for 30 minutes at 30 °C. Fluorescence intensity units were plotted against time to generate a linear reaction progress curve, where the initial velocity (V_0_) corresponds to the gradient of the curve (typically achieved within 15 minutes). IC_50_ values were calculated using the GraphPad Prism (v9.0) built-in dose-response equation for inhibitor concentration vs. response (variable slope).

### In-gel polyubiquitin cleavage assays and immunoblotting

The inhibitor capzimin (Merck, Germany) was diluted in 1 mM DTT and incubated for 30 minutes at room temperature to reduce disulphide bonds required for inhibitor activity. 1 nM BRISC(FL) was then incubated with either 10 or 100 μM inhibitor (FX-171-C, JMS-175–2, AP-5–144, thiolutin or capzimin) for 15 minutes at room temperature in DUB reaction buffer containing 50 mM HEPES-NaOH pH 7.0, 100 mM NaCl, 0.1 mg/mL BSA, 1 mM DTT, and 0.03% v/v Brij-35. 1 μM K63-linked tetraubiquitin chains (Boston Biochem, USA) were added and incubated with BRISC-inhibitor mixes for 30 minutes at room temperature. To analyse dose-dependent reduction in tetraubiquitin chain cleavage, 0.5 nM BRISC(FL) was incubated with 0–100 μM FX-171-C for 15 minutes. 500 nM tetraubiquitin chains (Boston Biochem, USA) were added and incubated for a further 30 minutes. Immunoblotting was performed with a commercially available HRP-conjugated antibody to Ub (Santa Cruz Biotechnology, USA).

### Mass photometry

For single-point measurement of BRISC and inhibitors, 1 μM BRISC(FL) was mixed with either DMSO, JMS-175–2, or FX-171-C at 330 μM and incubated for 15 minutes on ice. Immediately prior to mass photometry (MP) measurement, the BRISC-inhibitor mix was diluted in 25 mM HEPES pH 7.5, 150 mM NaCl, 1 mM TCEP to a final concentration of 10 nM BRISC (0.05% v/v DMSO). 12 μL of the diluted sample were used for the final MP measurement, following autofocus stabilisation. For measurements with increasing concentrations of JMS-175–2 and FX-171-C, 2-fold dilutions of inhibitor in 100% v/v DMSO generated a dilution series with concentrations of inhibitor from 800 μM to 0 μM. 0.5 μL inhibitor was mixed with 19.5 μL 50 nM BRISCDNDC (2.5% v/v DMSO) and incubated at room temperature for 15 minutes. The BRISC-inhibitor mix was used directly for MP measurement using buffer-free autofocus stabilisation.

Microscope coverslips were prepared as previously described^62^. All mass photometry experiments were performed using a One^MP^ mass photometer (Refeyn). Movies were recorded for 60 seconds using AcquireMP (Refeyn), and were processed using DiscoverMP (Refeyn). Mass photometry image processing has been previously described^62^. Briefly, contrast-to-mass (C2M) calibration was performed using protein standards (66–669 kDa) diluted in gel filtration buffer. The output from each individual movie resulted in a list of particle contrasts which were converted to mass used the C2M calibration. The mass distribution from each run is in a histogram, where count refers to each landing event and a Gaussian sum is fitted to the data. The relative amount of each species is calculated as the area of each Gaussian, where σ refers to the standard deviation of the fitted Gaussian. Dimer fraction refers to the percentage of the total counts which correspond to the 564 kDa BRISCΔNΔC dimer complex. Curves EC_50_ values were fitted and calculated using the GraphPad Prism (v9.0) built-in dose-response equation for concentration of agonist vs. response (variable slope).

### Negative stain electron microscopy – grid preparation, data collection and image processing

BRISC(FL) or *Mm*BRISC(FL) was mixed with inhibitor (or DMSO) on ice for 30 minutes at a final concentration of 1 μM BRISC and 100 μM inhibitor. The BRISC-inhibitor mix was diluted in gel filtration buffer to a final concentration of 0.014 mg/mL (0.5% v/v DMSO). Sample was immediately loaded onto carbon-coated copper grids (Formvar/Carbon 300 mesh Cu, Agar Scientific). Grids were glow discharged for 30 seconds, at 10 mA, and 0.39 mBar pressure (PELCO easiGlow, Ted Pella). Grids were incubated for 1 minute with 7 μL sample, washed three times with H_2_O, stained twice with 2% w/v uranyl acetate for a total of 30 seconds. Excess liquid was removed by blotting with filter paper. Data were collected using an FEI Tecnai F20 microscope at 200 KeV, fitted with an FEI CETA (CMOS CCD) camera. Micrographs were collected at 29000x magnification with a pixel size of 3.51 Å. RELION (v3.0 and v3.1) were used for processing of negative stain EM data^63,64^. Approximately, 2000 particles were manually picked and extracted with a box size of 128 Å^2^. These particles were used for reference-free 2D class averaging to generate 2D templates for autopicking. The parameters for autopicking were optimised and between 5000–10000 particles were extracted. Two rounds of 2D classification were used to remove junk particles and assess the stoichiometry of the BRISC complex.

### Cryo-electron microscopy grid preparation and data collection

For all the BRISC(FL) and BRISC-JMS-175–2 cryo-EM datasets, Quantifoil R1.2/1.3 300 mesh copper grids were glow-discharged using a GloQube (Quorum) for 30 seconds at 40 mA. BRISC(FL) was loaded directly onto grids at 0.4 mg/mL. BRISCDNDC at 0.3 mg/mL (2 μM) was mixed with JMS-175–2 at 200 μM in buffer (25 mM HEPES pH 7.5, 150 mM NaCl, 1 mM TCEP) for 30 minutes on ice. For BRISC-FX-171-C grids, BRISCDNDC at 0.7 mg/mL (5 μM) was mixed with FX-171-C at 400 μM, and loaded onto grids which were plasma cleaned in downstream mode at radio-frequency power 43 W for 30 seconds using a Tergeo plasma cleaner (Pie Scientific). An FEI Vitrobot IV (ThermoFisher) was equilibrated to 4 °C at 100% relative humidity. Grids were blotted at blot force 3 for 4 seconds and plunged into liquid ethane cooled by liquid nitrogen for vitrification.

Micrograph movies were collected on a Titan Krios transmission electron microscope (ThermoFisher) at 300 keV. The BRISC(FL) dataset (1,610 movies) was collected on an FEI Falcon III direct electron detector in integrating mode using a final electron dose of 88 e^−^/Å^2 65^, a magnification of ×75,000, and a final calibrated object sampling of 1.065 Å/pixel. 1,610 movies were collected using EPU automated acquisition software. Each exposure movie was 1.5 s, collected over 59 fractions with a dose of 1.49 e^−^/Å^2^ per fraction. One exposure was taken per hole and the defocus values ranged from −1.6 μm to −3.1 μm.

The BRISC-FX-171-C dataset was collected on an FEI Falcon 4 direct electron detector (ThermoFisher) with a 10 eV Selectris energy filter (ThermoFisher). In total, 16,750 movies were collected using EPU automated acquisition software in counting mode. A dose per physical pixel per second of 5.14 resulting in a dose of 34.97 e^−^/Å^2^, fractionated across 118 EPU frames. These were grouped into 44 frames, resulting in a dose per frame of 0.8 e^−^/Å^2^, and a final calibrated object sampling of 0.71 Å/pixel. Three exposures were taken per hole, at a ×165,000 magnification, with the defocus values ranging from −1.6 μm to −2.5 μm. Detailed information in **Extended Data Table 1**.

The BRISC-JMS-175–2 dataset was collected on an FEI Falcon 4 direct electron detector (ThermoFisher). In total, 7,771 movies were collected using EPU automated acquisition software in counting mode. A dose per physical pixel per second of 4.92 resulting in a dose of 40.01 e^−^/Å^2^, fractionated across 204 EPU frames. These were grouped into 40 frames, resulting in a dose per frame of 0.99 e^−^/Å^2^, and a final calibrated object sampling of 0.82 Å/pixel. Three exposures were taken per hole, at a ×96,000 magnification, with the defocus values ranging from −1.7 μm to −3.1 μm.

### Image processing

Image processing was carried out using RELION (v.3.0 and v.3.1)^63,64^. For the BRISC(FL) dataset collected on the Falcon III detector, drift correction was performed using MotionCor2^66^ and real-time contrast transfer functions were estimated using gCTF^67^. For the BRISC + JMS-175–2/FX-171-C datasets collected using the Falcon 4 detector, motion was corrected using RELION’s own implementation of the MotionCor2 algorithm^68^; the Contrast Transfer Function (CTF) was estimated for the BRISC-FX-171-C dataset using CTFFIND^69^, and for the BRISC-JMS-175–2 dataset using gCTF^67^. Motion correction and CTF estimation were performed on-the-fly^70^.

For the BRISC(FL) dataset, particles were picked using crYOLO (v.1.3.5)^71^ using a model trained on 10 micrographs. Particles coordinates were imported into RELION (v3.0) and 282,442 particles were extracted with a box size of 350 pixels. Data processing is summarised in **Extended Data Fig. 3b**. Particles were subjected to two rounds of reference-free 2D classification in both RELION and cryoSPARC (v3.3.1). For the BRISC only structure, classes corresponding to monomeric BRISC (117,891 particles) were selected for cryoSPARC *ab initio* reconstruction. The best class (50,083 particles) was selected and subjected to homogeneous refinement followed by non-uniform refinement with defocus and global CTF refinement applied. The resulting map was resolved to 8.1 Å, and a BRISC structure with SHMT2 removed (PDB: 6H3C) was rigid body fitted using Chimera (v1.12)^72^ and visualised using ChimeraX (v1.2.3)^73^. To process the new BRISC dimer conformation, RELION 2D class averages (6,344 particles) corresponding to a BRISC dimer were selected to generate a reference model for 3D classification. Two classes were selected (3,244 particles) for 3D refinement. BRISC structures with SHMT2 removed (PDB: 6H3C) were rigid body fitted using UCSF Chimera (v1.12)^72^ and visualised using ChimeraX (v1.2.3).

Data processing for the BRISCDNDC-FX-171-C dataset is outlined in **Extended Data Fig. 4b**. Briefly, a model was trained using crYOLO (v.1.6.1)^71^ using particles picked from 10 micrographs, and this model was used to pick 2,458,785 particles which were imported and extracted using RELION (v3.1.1) Particles were binned by 2 and extracted with a box size of 192 pixels. Particles were subjected to one round of reference-free 2D classification. A BRISC-JMS-175–2 map was low-pass filtered and used as an initial model for 3D classification with no symmetry applied. The two best classes (632,988 particles) were selected for 3D refinement, post-processing and three rounds of particle polishing and CTF refinement, resulting in a final map at 3.02 Å. To improve the density around the small molecule binding site a mask was applied during refinement to one half of the map **(Extended Data Fig. 4g)**. The resolution of the map improved to 2.8 Å and 2.7 Å around the BLUE binding site.

A schematic **(Extended Data Fig. 4e)** details the data processing pipeline for the BRISCΔNΔC-JMS-175–2 dataset. In summary, particle picking was performed using crYOLO (v.1.6.1)^71^. A model was trained from manually picking 14 micrographs. The trained model picked 1,616,457 particles, for which the coordinates were imported into RELION (v3.1.1) for extraction with 2x binning and a box size of 176 pixels. Two rounds of reference-free 2D classification was used to remove junk particles. A reference model from a previous BRISC-JMS-175–2 dataset was applied during 3D classification of 1,011,924 particles with no symmetry applied. 371,872 particles were selected from three classes and re-extracted with a box size of 352 pixels. After 3D refinement and post-processing, a reconstruction of a BRISC dimer complex was achieved at 3.98 Å. Iterative rounds of per-particle contrast function refinement and Bayesian polishing produced an improved final map at 3.32 Å. To further improve the density around the small-molecule binding site, a mask was applied during 3D refinement, encompassing only the better resolved half of the map **(Extended Data Fig. 4i)**. This improved the density for “half” of the structure, resulting in a 3.2 Å map. Final resolutions were determined using the gold-standard Fourier shell correlation criterion (FSC = 0.143). Local resolution estimation was carried out using the RELION local resolution feature.

### Model building and refinement

Atomic models of the BRISC dimer in complex with either JMS-175–2 or FX-171-C were built using high resolution cryo-EM maps. A preliminary model of the human BRCC36-Abraxas2 super dimer was acquired from our previous BRISC-SHMT2 model (PDB: 6R8F)^20^ with BRCC45 and SHMT2 removed. The BRCC36-Abraxas2 super dimer was rigid-body fitted into the cryo-EM density using UCSF Chimera^72^ and manually modelled into the BRISC-FX-171-C map using Coot^74,75^. The super dimer was duplicated, rigid body fitted, and manually modelled into the BRCC36-Abraxas2 in the opposite side of the map. A model for human BRCC45 and MERIT40 was acquired from a previous BRISC-SHMT2 model (PDB: 6H3C)^21^ and rigid body fitted into the cryo-EM density. The BRCC45 N-termini (residues 1–275) were manually modelled using Coot, but due to the lower resolution of the map beyond the UEV-M domain and for MERIT40, these regions were rigid body fitted into the density based on previous BRISC-SHMT2 structures^20,21^. The BRCC45-MERIT40 arms were duplicated, rigid body fitted, and modelled into the density corresponding to the second BRISC molecule. The side chain atoms for BRCC45 (residues 275–383) and MERIT40 were set to zero occupancy due to lower resolution of the EM maps in these regions. Small molecule chemical structures were generated in ChemDraw (PerkinElmer), and PDB and CIF files were created using the PRODRG2 server^76^ or eLBOW^77^. FX-171-C compounds were manually fit into the density using UCSF Chimera, and refined using COOT Real Space Refine. The model was refined against the BRISC-FX-171-C map using Phenix real-space refinement (v1.20)^78^. To build the BRISC-JMS-175–2 structure, FX-171-C was removed from the model and replaced with JMS-175–2. The model was rigid body fitted into the density for a BRISC-JMS-175–2 cryo-EM map and subjected to iterative rounds of manual building in Coot. The BRISC-JMS-175–2 model was refined using Phenix real-space refinement (v1.20).

### Sequence alignments, structure visualisation, and analysis

Multiple sequence alignments were peformed using MUSCLE^79^ and edited using ALINE (v1.0.025)^80^. Electron microscopy maps and structure models were visualised in UCSF Chimera (v1.12.0)^72^ and ChimeraX (v1.2.3)^73^.

### Native mass spectrometry

BRISC(FL) at 10 μM was mixed with 1 mM inhibitor (JMS-175–2 or FX-171-C) or DMSO (2.5%) and incubated on ice for 30 minutes. Samples were buffer exchanged into 500 mM ammonium acetate using Zeba Spin 7K MWCO desalting columns (ThermoFisher). Samples were analysed by nanoelectrospray ionisation MS using a quadrupole-orbitrap MS (Q-Exactive UHMR, ThermoFisher Scientific) using gold/palladium coated nanospray tips prepared in-house. The MS was operated in positive ion mode using a capillary voltage of 1.5 kV, capillary temperature of 250 °C and S-lens RF of 200 V. In-source trapping was used with a desolvation voltage of −200 V for 4 μs. Extended trapping was not used. The quadrupole mass range was 2000–15000 m/z. Nitrogen gas was used in the HCD cell with a trap gas pressure setting of 5. Orbitrap resolution was 6250, detector *m/z* optimisation was low. Five microscans were averaged and an AGC target of 2 ×10^5^ was used. Mass calibration was performed by a separate injection of sodium iodide at a concentration of 2 μg/μL. Data processing was performed using QualBrowser (v4.2.28.14) and deconvoluted using UniDec^81^.

### Hydrogen-deuterium exchange mass spectrometry

HDX-MS experiments were carried out using an automated HDX robot (LEAP Technologies, USA) coupled to an M-Class Acquity LC and HDX Manager (Waters, UK). For differential HDX-MS of BRISC in the absence and presence of inhibitor, 5 μM BRISCΔNΔC was mixed with 500 μM FX-171-C or DMSO (2.5% v/v) in buffer containing 25 mM HEPES pH 7.5, 150 mM NaCl, 1 mM TCEP. The BRISC-inhibitor or BRISC-DMSO mix was incubated on ice for 30 minutes prior to deuterium exchange reactions. For labelling, 5 μL BRISC-inhibitor/DMSO mix was diluted in 95 μL deuterated buffer (50 mM potassium phosphate, 200 mM NaCl, pH 7.5) and incubated at 4°C for 0 seconds, 0.5, 1, 10 or 60 minutes. The sample was quenched by adding quench buffer (50 mM potassium phosphate, pH 2.1) at a 1:1 ratio and dropping the temperature to 0 °C. 50 μL of quenched sample was passed through an immobilised pepsin column (AffiPro, Czech Republic) at 115 μL min^−1^. and trapped on a VanGuard Pre-column Acquity UPLC BEH C18 (1.7 μm, 2.1 mm × 5 mm, Waters Ltd., Wilmslow, Manchester, UK) for 3 min in 0.3% v/v formic acid in water. The resulting peptic peptides were transferred to a C18 column (75 μm × 150 mm, Waters Ltd., Wilmslow, Manchester, UK) and separated by gradient elution of 0–40% MeCN (0.1% v/v formic acid) in H2O (0.3% v/v formic acid) over 7 min at 40 μl min^−1^. Trapping and gradient elution of peptides was performed at 0 °C. The HDX system was interfaced to a Synapt G2Si mass spectrometer (Waters Ltd., Wilmslow, Manchester, UK). HDMSE and dynamic range extension modes (Data Independent Analysis (DIA) coupled with IMS separation) were used to separate peptides prior to CID fragmentation in the transfer cell. HDX data were analysed using PLGS (v3.0.2) and DynamX (v3.0.0) software supplied with the mass spectrometer. Restrictions for identified peptides in DynamX were as follows: minimum intensity: 10000, minimum products per MS/MS spectrum: 3, minimum products per amino acid: 0.3, maximum sequence length: 18, maximum ppm error: 10, file threshold: 8/9. Following manual curation of the data, Woods and individual uptake plots were generated using Deuteros 2.0^82^. A summary of the HDX-MS data, as recommended by reported guidelines^83^, is shown in **Extended Data Table 2**.

### Cell lysis, immunoprecipitation and immunoblotting

Immortalised breast epithelial cell line MCF10A were cultured in 10 cm plate with DMEM + F12 (1:1) supplemented with 5 % horse serum, 100 ng/mL cholera toxin, 10 μg/mL insulin, 20 ng/mL epidermal growth factor (EGF) and 0.5 μg/mL hydrocortisone until >80% confluence. 120,000 cells were then seeded into 6cm plates. Cells were left to reach ultra-confluence. Once confluent, cells were washed with PBS and starved for 30 minutes in 2 mL DMEM+ F12 (1:1) supplemented with 1% horse serum. After 30 minutes cells were pre-treated with either DMSO (0.1 %) (Control and IFN only) or BRISC inhibitor (2.5 μM) for 15 minutes. After 15 minutes, hIFN2α (75 ng/mL) was added to the media and mixed it well. Cells were treated for indicated time or otherwise 1 hr (if BRISC inhibitor not used, 4hr if BRISC inhibitors used). Cells were washed with ice cold PBS and scraped with cell scrapers into PBS and centrifuged at 1200 rpm for 10 mins. Cell pellets were either resuspended into RIPA buffer supplemented with complete EDTA free protease inhibitor cocktail and 25 U/mL benzonase for immunoblotting or in immunoprecipiation buffer (100 mM NaCl, 0.2% Igepal CA-630, 1mM MgCl_2_, 10% glycerol, 5 mM NaF, 50 mM Tris-HCl, pH 7.5), supplemented with complete EDTA free protease inhibitor cocktail and 25 U/mL benzonase for co-immunoprecipitation. Lysates were mixed with 2X sample buffer for gel loading prior to Western blot analysis. Co-immunoprecipitation was performed using Anti-Flag M2 Affinity Gel beads and eluted with 0.2 M Glycine.

### Real-time quantitative PCR

Immortalised breast epithelial cell line MCF10A were cultured in 10 cm plates with DMEM + F12 (1:1) supplemented with 5 % horse serum, 100 ng/mL cholera toxin, 10 μg/mL insulin, 20 ng/mL epidermal growth factor (EGF) and 0.5 μg/mL hydrocortisone until >80% confluence. 100,000 cells were seeded into 6 well plates. Cells were left to reach ultra-confluence. Once confluent, cells were washed with PBS and starved for 30 minutes in 2 mL DMEM + F12 (1:1) supplemented with 1% horse serum. After 30 minutes cells were pre-treated with either DMSO (0.1%) (Control and IFN only) or BRISC inhibitor (2.5 μM) for 15 minutes. After 15 minutes, hIFN2α (75 ng/mL) was added to the media and mixed. Cells were treated for 4 hr. Cells were washed with ice cold PBS and scraped with cell scrapers into 350 μL RNAlater buffer and RNA was isolated using RNeasy mini kit and treated with on column DNase. Isolated RNA was quantified using a Nanodrop spectrophotometer. RNA was converted into cDNA and the complementary DNA was used for real-time quantitative PCR analysis of expression of interferon stimulated genes (ISG15, IFIT1, IFIT2, IFITM1, CXCL10 and 18s rRNA) using an Applied Biosystems Quantstudio 6 RT-PCR system. All experiments were carried out in triplicate.

### PBMCs *in vitro* experiments

Peripheral blood mononuclear cells (PBMCs) from healthy and SSc donors were separated using density gradient method (Leucosep^™^, Greiner Bio-One International) from EDTA anticoagulated peripheral blood; isolated cells were washed twice by PBS. PBMCs were then incubated in RPMI1640 media 10% FBS 1% PS (Gibco Laboratories) with DMSO (0.1%) (CTR) or in media containing JMS-175–2, FX-171-C, or AP-5–145 at 2 μM for 16 hours. For healthy PBMCs, cells were stimulated with 20 ng/mL of IFN (+/− compounds or DMSO). Cells were centrifuged at 300g for 10 minutes. Supernatant was removed and subjected to R&D Systems DY266 Human CXCL10/IP-10 DuoSet ELISA (Biotechne, Abingdon, UK) in duplicates, according to manufacturer’s guidance. Concentration of CXCL10 was determined according to a 4-parameter logistic regression standard curve. RNA was extracted from cell pellets using TRIzol^™^ (Thermo Fisher Scientific, Waltham, MA) and processed using Quick-RNA Miniprep Kit (Zymo Research, Irvine, CA) as per the manufacturer’s instruction.

### Gene expression analysis

For CXCL10 and composite IFN score gene expression analysis, RNA was reverse transcribed using the high-capacity cDNA synthesis kit (Applied Biosystems). qRT-PCR were performed using SyBr Green PCR kit (Thermo) with primers specific for CXCL10 (Forward; TCCAGTCTCAGCACCATGAA Reverse; AGGTACTCCTTGAATGCCACT), MX1 (Forward; CGACACGAGTTCCACAAATG Reverse; AAGCCTGGCAGCTCTCTACC), IFIT1 (Forward; GACTGGCAGAAGCCCAGACT Reverse; GCGGAAGGGATTTGAAAGCT), ISG15 (Forward; GTGGACAAATGCGACGAACC Reverse; ATTTCCGGCCCTTGATCCTG), and GAPDH (Forward; ACCCACTCCTCCACCTTTGA Reverse; CTGTTGCTGTAGCCAAATTCGT). The data obtained was analysed according to the ΔΔ Ct method relative to GAPDH. For composite IFN score, fold change in gene expression in AP-5–145, JMS-175–2 and FX-171-C treated samples (of each gene CXCL10, MX1, IFIT1, ISG15) was calculated relative to each donor DMSO control (CTR). The composite score represents grouped analysis of combined fold changes for all 4 genes.

For Type I IFN signalling analysis (67 genes), RNA was converted to cDNA using RT2 First Strand Kit (Qiagen). Next, the cDNA was mixed with RT2 SYBR Green Mastermix (Qiagen, Venlo, Netherlands). The human IFN I RT2 Profiler PCR Array (Qiagen cat. no. PAMM-016ZE) was performed and relative expression determined using the ΔΔCT method and normalized for 4 housekeeping genes (ACTB, GAPDH, HPRT1, RPLP0) according to manufacturer’s guidance. Triplicate repeats were performed.

### Inclusion and ethics

All participants enrolled provided written informed consent according to a protocol approved by Medicine and Health Regulatory agency (STRIKE NRES-011NE to FDG, IRAS 15/NE/0211).

### Statistical analysis

Comparisons between conditions were conducted using paired student t-test. A one way ANOVA with Dunnett’s multiple comparisons test was used to compare JMS-175–2 and FX-171-C groups to the AP-5–145 negative control. Statistical significance was defined as a p-value less than 0.05 for all analyses. Data analysis was performed using GraphPad Prism (v9.5.1).

### Data availability

Cryo-EM maps have been deposited in the Electron Microscopy Data bank under the accession codes EMD-17980 and EMD-18009. Model coordinates have been deposited in the Protein Data Bank under the accession codes 8PVY, 8PY2. HDX data are available via ProteomeXchange (identifier: PXD044584). All unique reagents are available upon request.

## Supplementary Material

Supplement 1

Supplement 2
