# Supplementary material for: Molecular glues that inhibit specific Zn2+-dependent DUB activity and inflammation
Source: bioRxiv. 2024 Sep 7:2024.09.07.611787. Preprint. [Version 1] doi: 10.1101/2024.09.07.611787 (PMC11398498; doi:10.1101/2024.09.07.611787)
Supplement: Supplementary file 1 [file media-1.pdf]

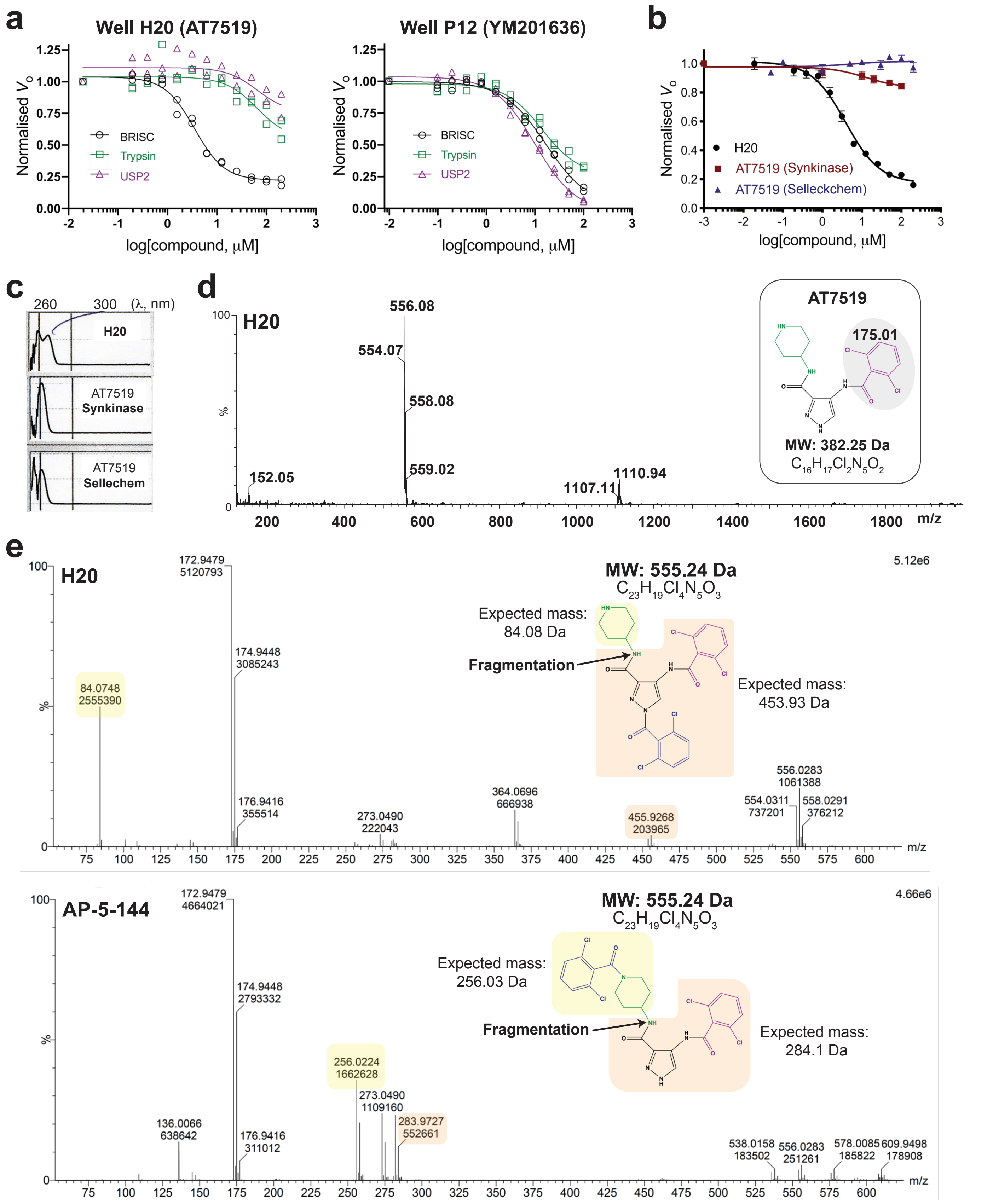

Extended Data Figure 1. Validation of hit compounds

a, Dose response curves for hit compounds against BRISC (1 nM), USP2 (100 nM) and trypsin (125 nM) using the internally-quenched fluorescence di-ubiquitin assay described in Fig. 1a. Data points from two experimental replicates are plotted. **b,** Re-testing of purchased H20 hit compound presumed to be AT7519. Data points are mean ± SEM from three independent experiments. **c,** UV-vis profile of compound in well H20 and purchased AT7519 compounds from Synkinase and Selleckchem.
d, Liquid-chromatography mass spectrometry (LC-MS) spectra of H20 compound. Insert, AT7519 structure. The difference between the H20 compound and AT7519 is 173 Da, which corresponds to the mass of a dichlorobenzaldehyde group. **e,** MS fragmentation analyses for H20 compound and a synthesised isomer, AP-5-144.

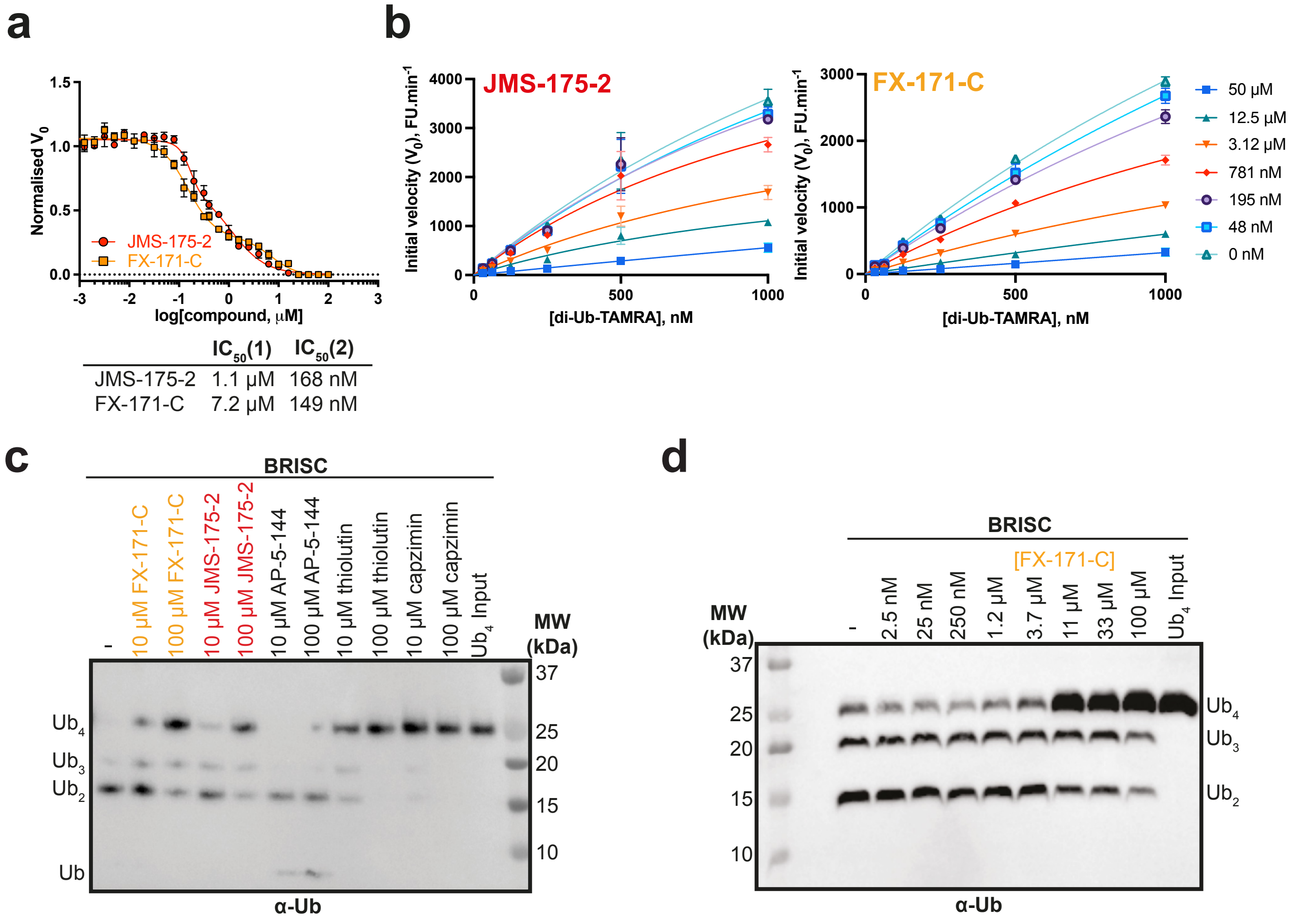

Extended Data Figure 2. BRISC inhibitors are non-competitive inhibitors and inhibit cleavage of longer ubiquitin chains

a, 32-point dose-response inhibition assay with JMS-175-2 and FX-171-C, with a biphasic curve fitted. Data points are mean ± SEM of two independent experiments carried out in duplicate.

b, Michaelis-Menten plots of BRISC activity against a K63-linked di-ubiquitin fluorogenic substrate with increasing concentrations of JMS-175-2 and FX-171-C. Data points are mean ± SEM of three independent experiments carried out in duplicate. **c,** Immunoblot analysis of BRISC-mediated tetraubiquitin cleavage in the absence and presence of 10 μM or 100 μM compound. **d,** Immunoblot analysis of BRISC-mediated K63-linked tetraubiquitin cleavage at increasing concentrations of FX-171-C.

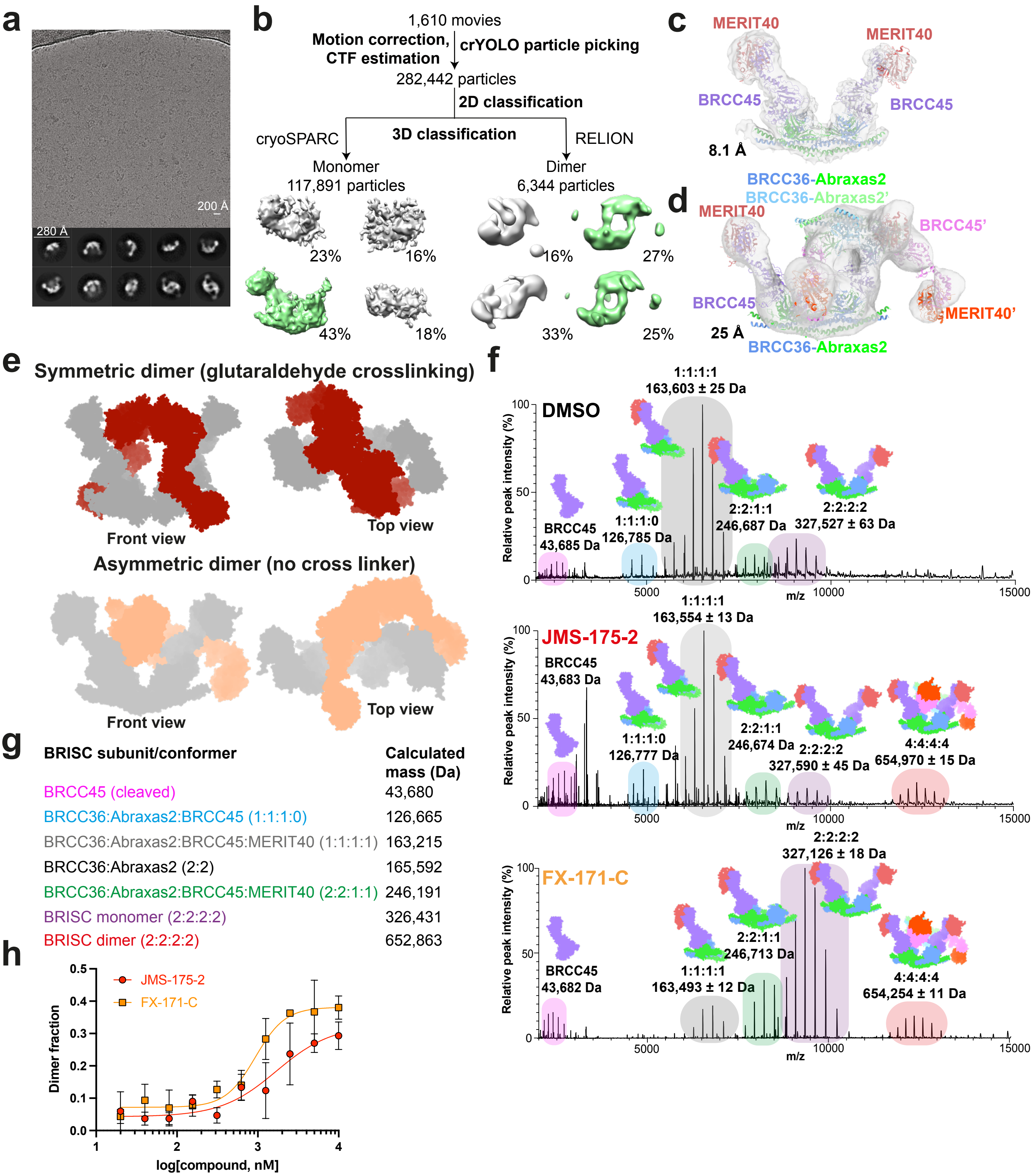

BRISC subunit/conformer	Calculated mass (Da)
BRCC45 (cleaved)	43,680
BRCC36:Abraxas2:BRCC45 (1:1:1:0)	126,665
BRCC36:Abraxas2:BRCC45:MERIT40 (1:1:1:1)	163,215
BRCC36:Abraxas2 (2:2)	165,592
BRCC36:Abraxas2:BRCC45:MERIT40 (2:2:1:1)	246,191
BRISC monomer (2:2:2:2)	326,431
BRISC dimer (2:2:2:2)	652,863

Extended Data Figure 3. Identification of a higher-order BRISC conformation
a, Representative micrograph from the BRISC only dataset (top), and 2D class averages (bottom). **b,** Cryo-EM processing workflow for BRISC monomer and dimer. Green indicates selected classes for 3D refinement. **c,** Cryo-EM density map (50,883 particles) with BRISC model (PDB: 6H3C) rigid body fitted. **d,** Low resolution cryo-EM density map of dimeric BRISC (3,244 particles), with two BRISC models rigid body fitted. **e,** Top, surface model of ARISC dimers observed in negative stain EM from grids prepared using the GraFix cross-linking method. The same conformation is reported for BRISC dimers from nsEM grids prepared using GraFix. Bottom, an asymmetric BRISC dimer conformation observed in cryo-EM without cross-linking. **f,** Native mass spectra of BRISC mixed with DMSO (control), JMS-175-2, or FX-171-C. BRISC complexes and subcomplexes are highlighted. **g,** Table of calculated masses for different BRISC subcomplexes and super complexes. **h,** Mass photometry measurements of BRISC dimer at increasing inhibitor concentration. Counts corresponding to BRISC dimer as a fraction of total counts are plotted. Error bars are ± SEM from three independent experiments.

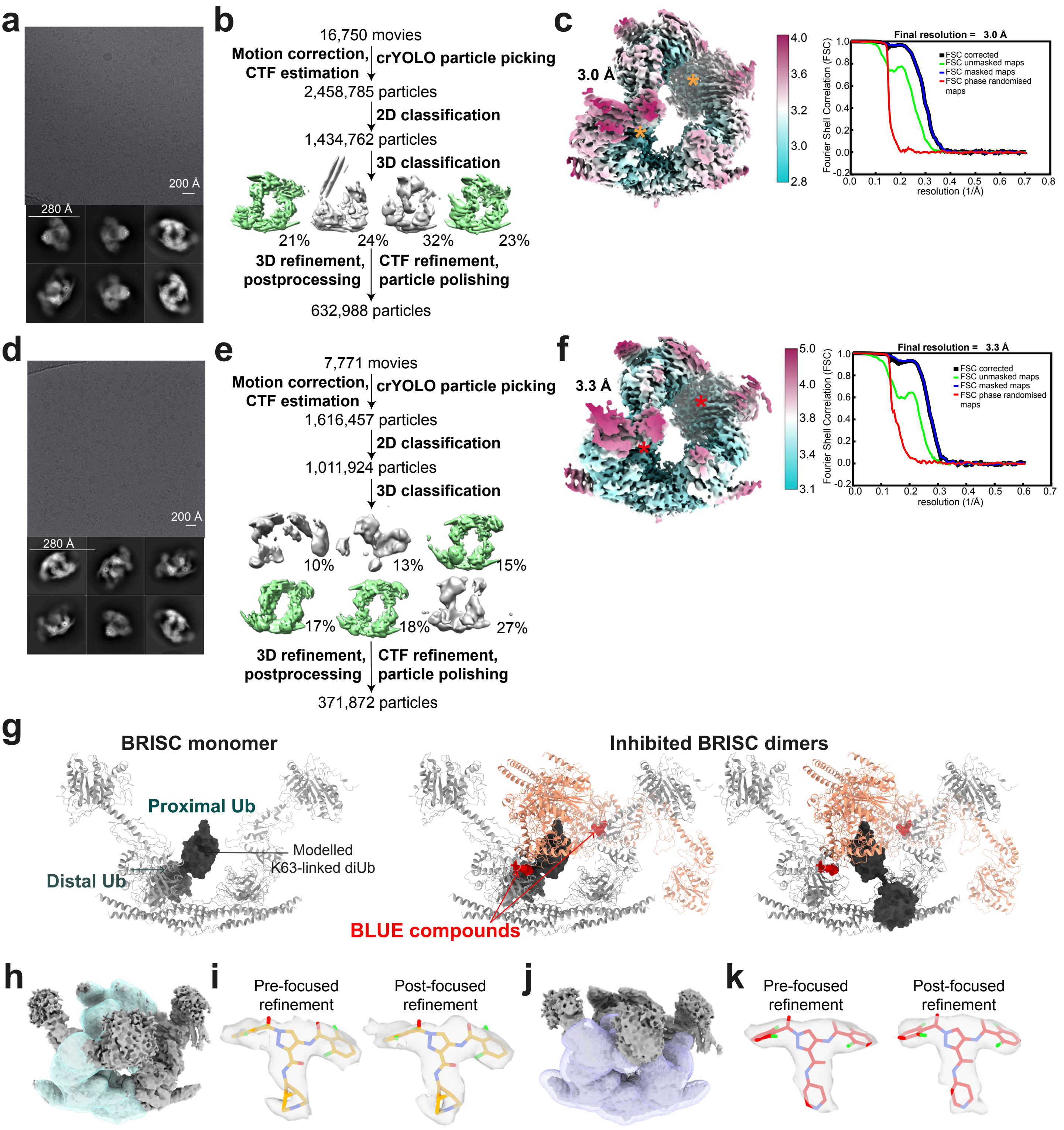

Extended Data Figure 4. Cryo-EM processing of the BRISC-inhibitor co-complex
Figures **a-c** correspond to the BRISC-FX-171-C cryo-EM dataset. Figures **d-f** correspond to the BRISC-JMS-175-2 dataset. **a, d,** Representative micrographs and 2D class averages. **b, e,** Image processing workflow. Green maps indicate selected classes used for 3D refinement. **c, f,** Cryo-EM density maps after 3D refinement and FSC curves for the final reconstructions used for model building. Asterisks indicate BLUE compound binding sites. Final resolution was calculated using the gold-standard FSC cut off at 0.143 frequency. **g,** K63-linked diUb (dark grey) modelled on the MPN^{+} domain of BRCC36 in BRISC (light grey), based on the AMSH LP-diUb structure (PDB: 2ZNV). Upon dimer formation, the second BRISC monomer sterically clashes with the proximal ubiquitin when it is bound to either BRCC36 active site. **h,** Mask used for focused refinement of the BRISC-FX-171-C map. **i,** Chemical structure of FX-171-C fitted into EM density before (left) and after (right) focused refinement. **j,** Mask applied during refinement of BRISC-JMS-175-2 map. **k,** Chemical structure of JMS-175-2 fitted into EM density before (left) and after (right) focused refinement.

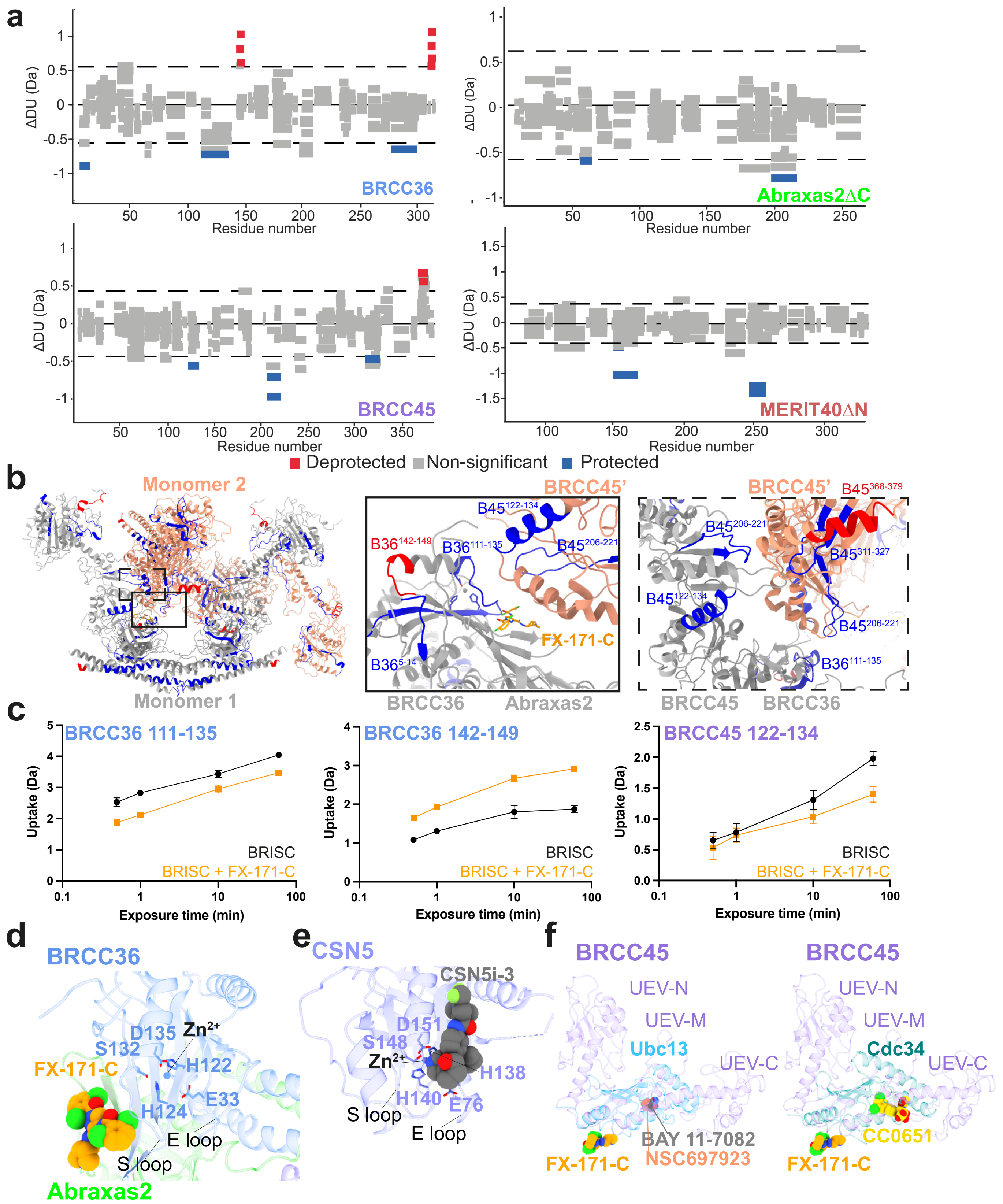

Extended Data Figure 5. Observed changes in BRISC subunit solvent accessibility and secondary structure in the presence of FX-171-C by HDX-MS

a, Wood's plots generated with Deuteros showing the differences in deuterium uptake over all four HDX timepoints, comparing BRISC in the absence and presence of FX-171-C. Regions highlighted in grey indicate peptides with no significant change, calculated using a 99% confidence interval, between the two conditions. The dashed line indicates the 99% confidence limit. Peptides are coloured in red to indicate deprotection in the presence of inhibitor, and blue to indicate protection. **b,** Peptides mapped onto BRISC dimer structure, highlighting peptides near BLUE binding site and at the interface of two BRCC45 subunits. B36 = BRCC36; B45 = BRCC45. **c,** Example deuterium uptake curves in the absence and presence of FX-171-C. Error bars are ± SEM from three technical replicates. **d,** BLUE compounds are allosteric inhibitors and do not disrupt the BRCC36 Zn^{2+} binding site. **e,** CSN5 active site in complex with inhibitor CSN5i-3 (PDB: 5J0G). **f,** Left, BRCC45 UEV-M bound to FX-171-C aligned to Ubc13 in complex with BAY 11-7082 (PDB: 4ONN) and NSC697923 (PDB: 4ONM). Right, BRCC45 UEV-M bound to FX-171-C aligned to Cdc34 in complex with CC0651 (PDB: 3RZ3).

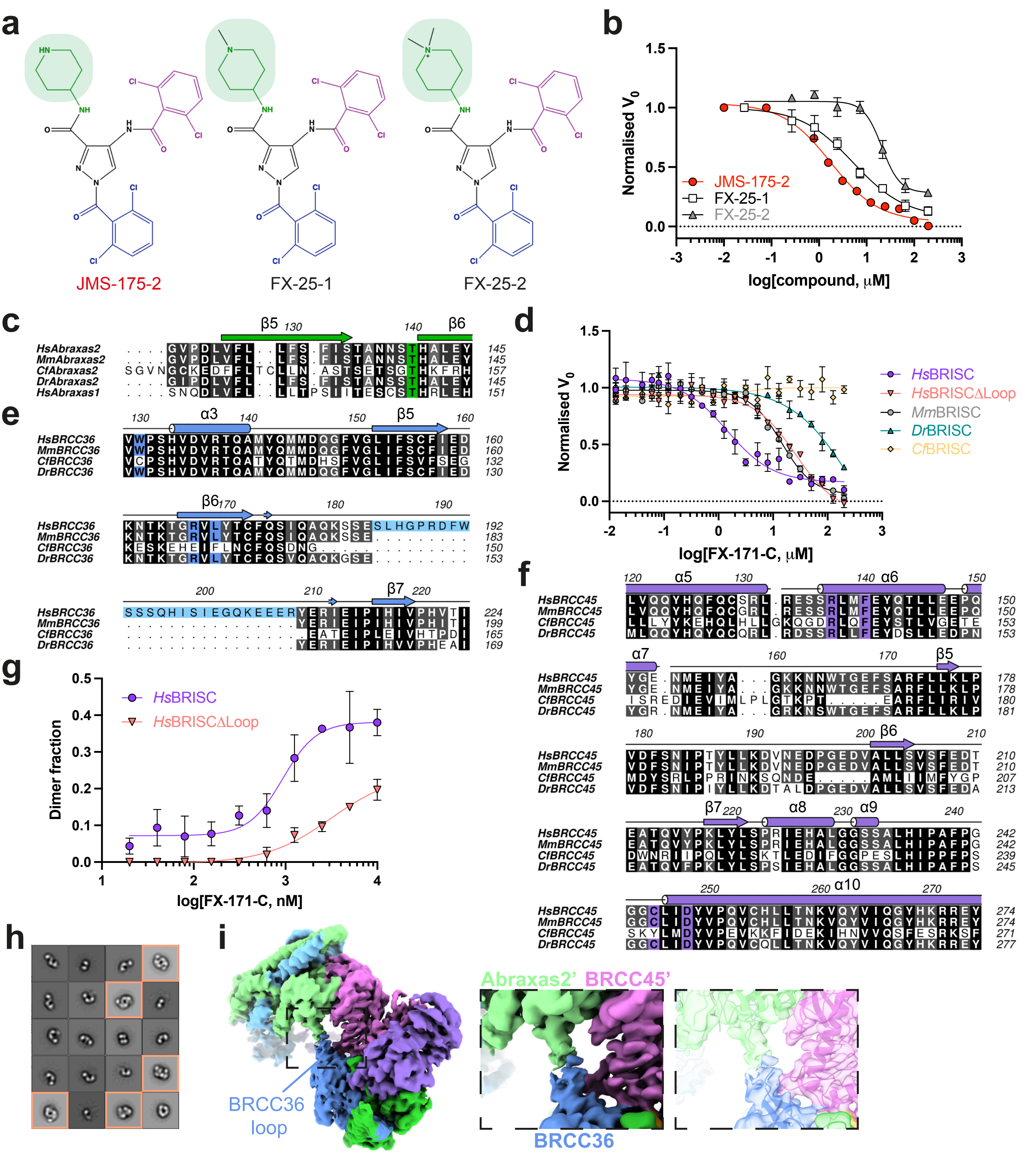

Extended Data Figure 6. BLUE compounds are allosteric inhibitors and selective for human BRISC
a, Chemical structure of JMS-175-2 and analogues FX-25-1, FX-25-2 which have substitutions in the piperidine ring (highlighted in green). **b,** Dose-response inhibition of BRISC by indicated compounds. IC$_{50}$ values: JMS-175-2 = 3.8 μM, FX-25-1 = 5.2 μM, FX-25-2 = 21 μM. Data points are mean ± SEM of three independent experiments carried out in duplicate. **c,** Multiple sequence alignment (black = conserved, white = not conserved) of Abraxas1 and Abraxas2 from indicated species. Coloured boxes indicate BLUE interacting residues. **d,** FX-171-C inhibition of different BRISC orthologues. *Hs* - *H. sapiens*, *Mm* - *M. musculus*, *Dr* - *D. rerio*, *Cf* - *C. floridanus*. Data points are mean ± SEM of three independent experiments carried out in duplicate. **e, f,** Multiple sequence alignment of **e,** BRCC36 and **f,** BRCC45 from indicated BRISC orthologues. **g,** Mass photometry analyses of dimer formation with FX-171-C for *Hs*BRISCΔNΔC and *Hs*BRISCΔLoop. Fraction of counts corresponding to BRISC dimer are plotted. Data points are mean ± SEM of three independent experiments. **h,** Negative stain EM 2D class averages of *Hs*BRISCΔLoop incubated with FX-171-C. 22% of particles in the 2D class averages correspond to BRISC dimers. **i,** BRISC-FX-171-C cryo-EM density map at threshold 0.0044 highlighting an extended loop in BRCC36.

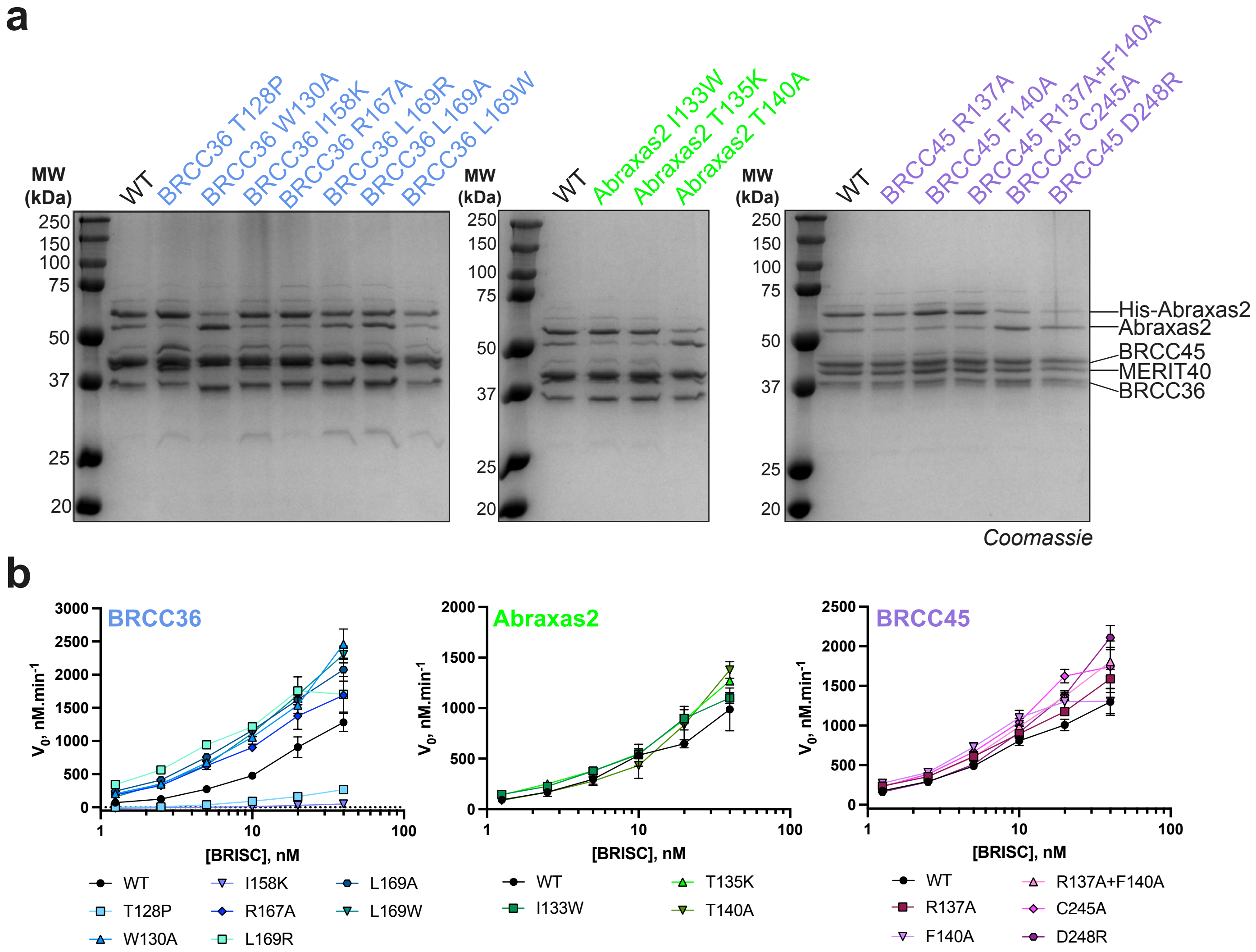

c

Mutation	FX-171-C IC_{50}
WT	616 nM
BRCC36 W130A	23 μM
BRCC36 R167A	1.4 μM
BRCC36 L169R	75 μM
BRCC36 L169A	33 μM
BRCC36 L169W	91 μM
Abraxas2 I133W	2.3 μM
Abraxas2 T135K	1.5 μM
Abraxas2 T140A	5 μM
BRCC45 R137A	-
BRCC45 F140A	4.3 μM
BRCC45 R137A+F140A	52 μM
BRCC45 C245A	31 μM
BRCC45 D248R	>100 μM

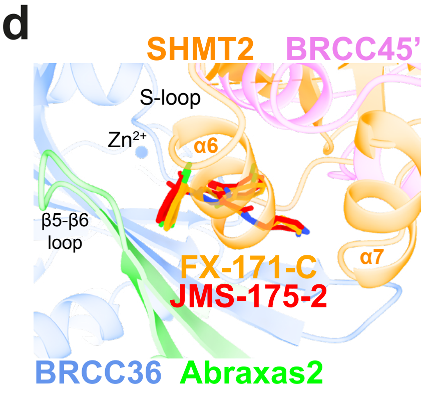

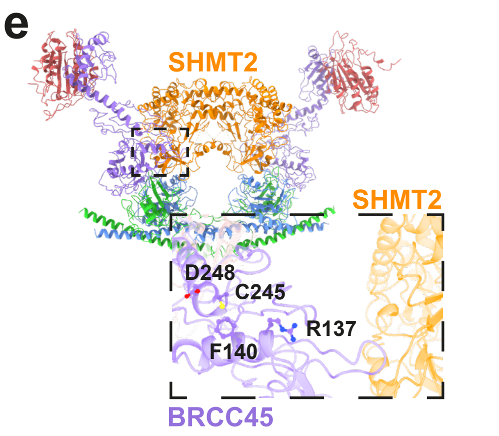

Extended Data Figure 7. Determining the DUB activity, inhibitor sensitivity, and SHMT2 inhibition of structure-guided mutants

a, SDS-PAGE analysis of purified BRISC mutants. **b,** Activity of BRISC mutants against an IQF di-ubiquitin substrate. Data points are mean ± SEM of three independent experiments carried out in duplicate. **c,** FX-171-C IC_{50} values from inhibition assays shown in **Figure 3**. **d,** Superimposition of SHMT2 dimer from BRISC-SHMT2 structure (PDB: 6R8F) onto BRISC-FX-171-C dimer structure. SHMT2 α6 helix clashes with the BLUE binding site. **e,** Mutated residues in BRCC45 are not in close proximity to SHMT2 binding site in the BRISC-SHMT2 structure (PDB: 6R8F).

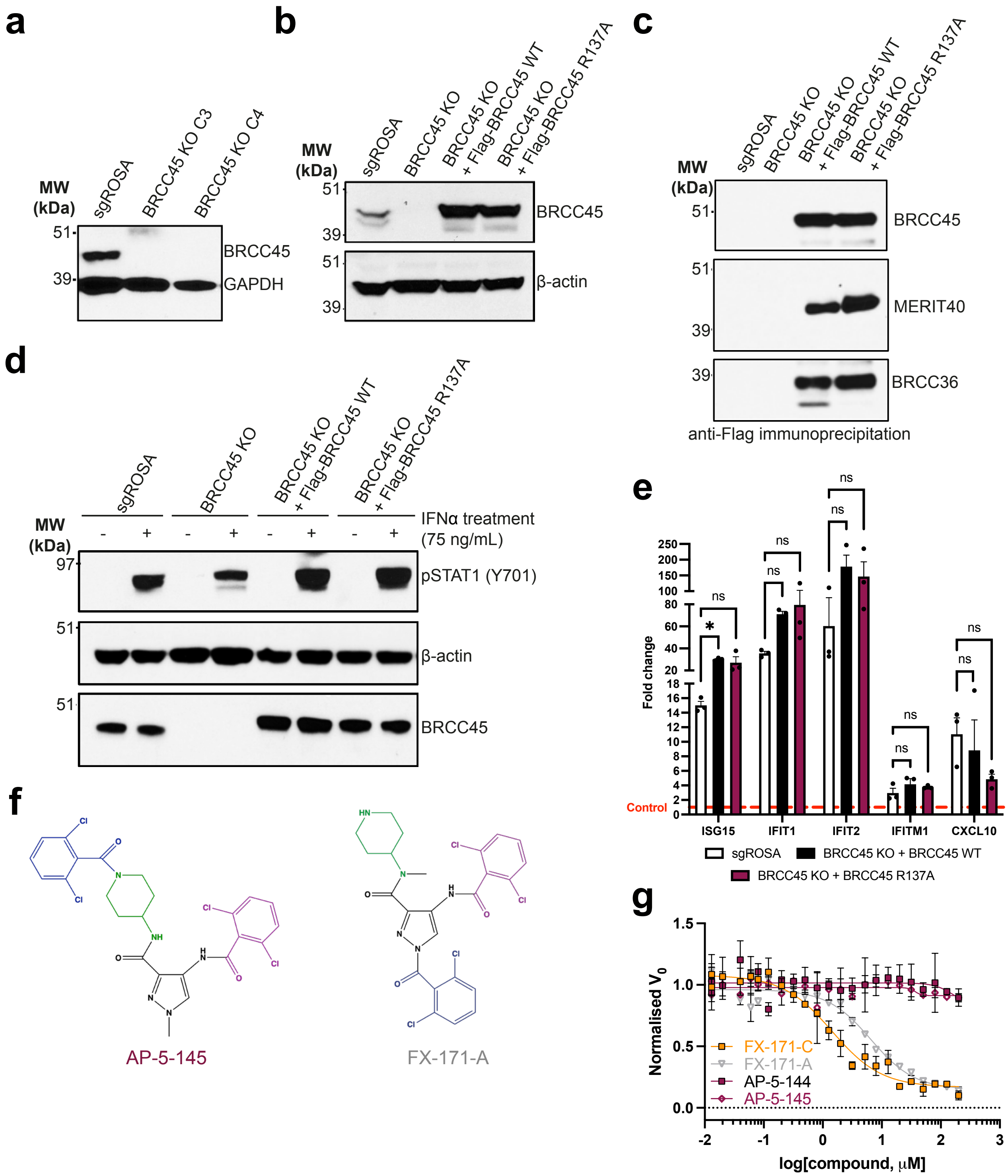

Extended Data Figure 8. Generating BRISC BRCC45 variants in MCF10A cells and establishing the effect of IFN stimulus

a, BRCC45 protein levels in selected clones after knock out in MCF10A Cas9 cells. sgROSA was used as a CRISPR Cas9 control. **b,** BRCC45 expression in whole cell lysates from MCF10A Cas9 cells expressing CRISPR control (sgROSA), BRCC45 WT, and BRCC45 R137A. **c,** Anti-Flag co-immunoprecipitation performed in indicated MCF10A cell lines. BRISC complex subunits were detected using specific antibodies. **d,** MCF10A cell lines were treated with and without hIFNα for 1 hour. STAT1 Tyr701 phopshorylation, BRCC45 and total protein levels (β-actin) were detected using specific antibodies. **e,** MCF10A Cas9 cells were treated with or without hIFNα for 4 hours. Expression of interferon-induced genes, ISG15, IFIT1, IFIT2, IFITM1, and CXCL10 were normalised to 18s rRNA and presented as fold change to own non-IFN treated control (n=3). A one-way ANOVA was used to compare statistical significance. Error bars are ± SEM. **f,** Chemical structures of compounds AP-5-145 and FX-171-A. **g,** Dose-response inhibition of BRISC by indicated compounds. Data points are mean ± SEM for three independent experiments.

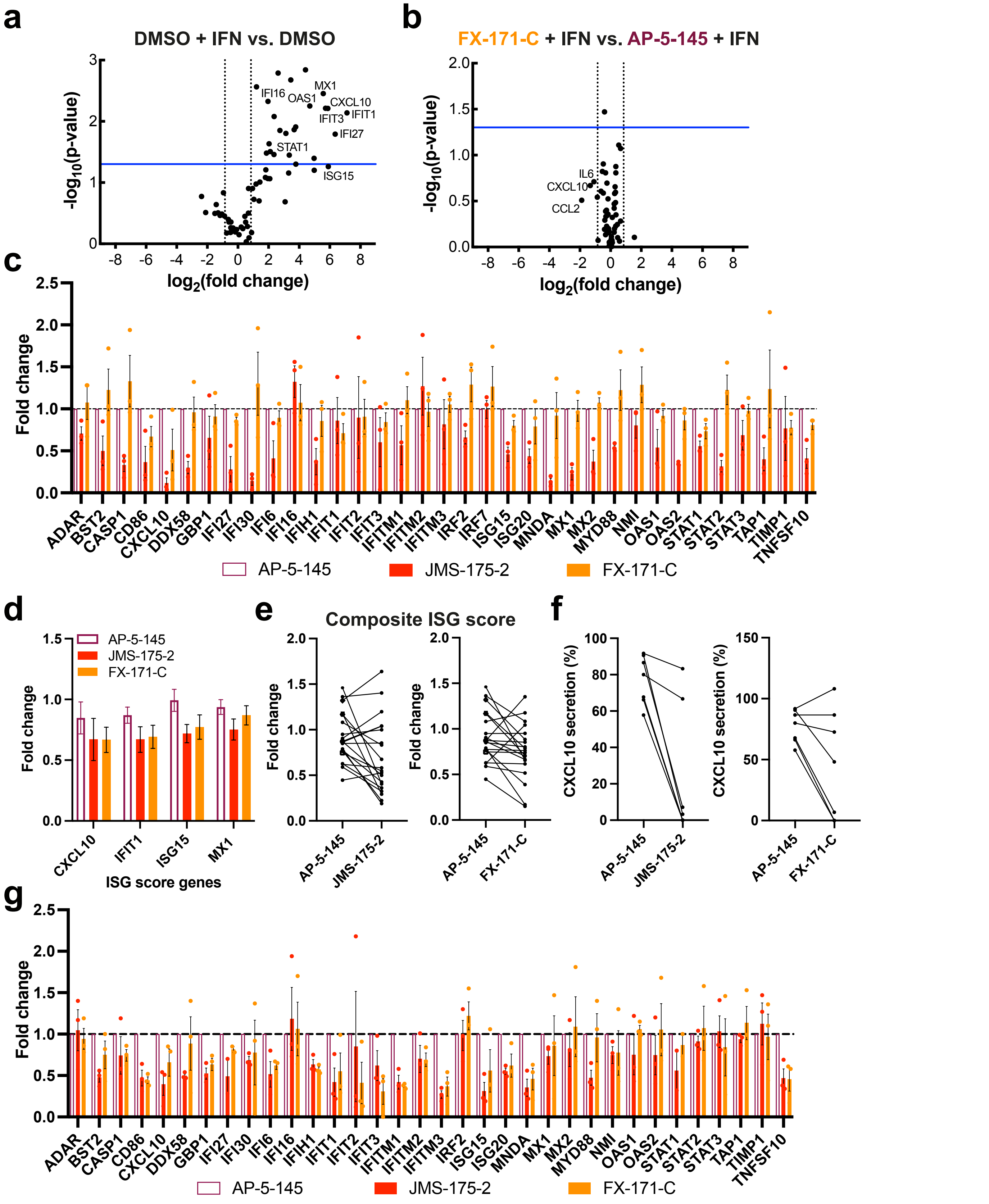

Extended Data Figure 9. BLUE compounds reduce interferon-stimulated gene expression in healthy and SSc PBMCs

a, Type I IFN signalling gene expression analysis (67 ISGs) in healthy control PBMCs treated with/without IFN (20 ng/mL) in DMSO control (0.1 %) for 16 h. Volcano plot illustrating genes increased with addition of IFN vs. DMSO only (n=3). **b,** As in **a,** healthy control PBMCs with 2 µM FX-171-C or AP-5-145 negative control. Volcano plot shows fold changes in gene expression with FX-171-C treatment vs. AP-5-145 condition (n=3). **c,** As in **a, b,** fold change in the expression of 35 ISGs with JMS-175-2 or FX-171-C treatment compared to AP-5-145 (n=3). One-way ANOVA Dunnett's multiple comparisons test: JMS-175-2 vs. AP-5-145 ****, FX-171-C vs. AP-5-145 ns. **d,** SSc PBMCs (n=20 donors) treated with DMSO, AP-5-145, FX-171-C, or JMS-175-2 for 16 h. Fold change in gene expression of the 4 ISGs in the composite ISG score. **e,** As in **d,** Composite ISG score gene expression analysis relative to each donors DMSO control. Changes in ISG score compared between conditions for each donor. **f,** ELISA analysis of CXCL10 protein secretion from SSc PBMCs supernatant (n=7). **g,** BRISC inhibitor modulated genes assessed using Type I IFN superarray gene expression analysis and fold change to each donors AP-5-145 condition for JMS-175-2 and FX-171-C (n=3). One-way ANOVA Dunnett's multiple comparisons test: JMS-175-2 vs. AP-5-145 ****, FX-171-C vs. AP-5-145 ****. Error bars are ± SEM.

a

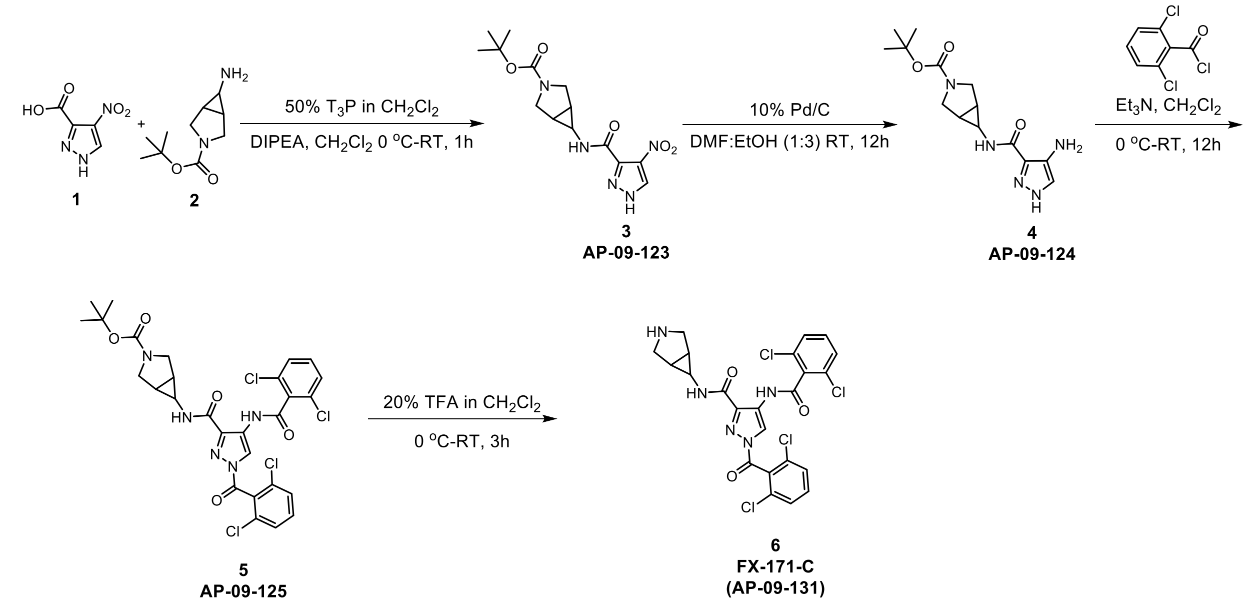

b

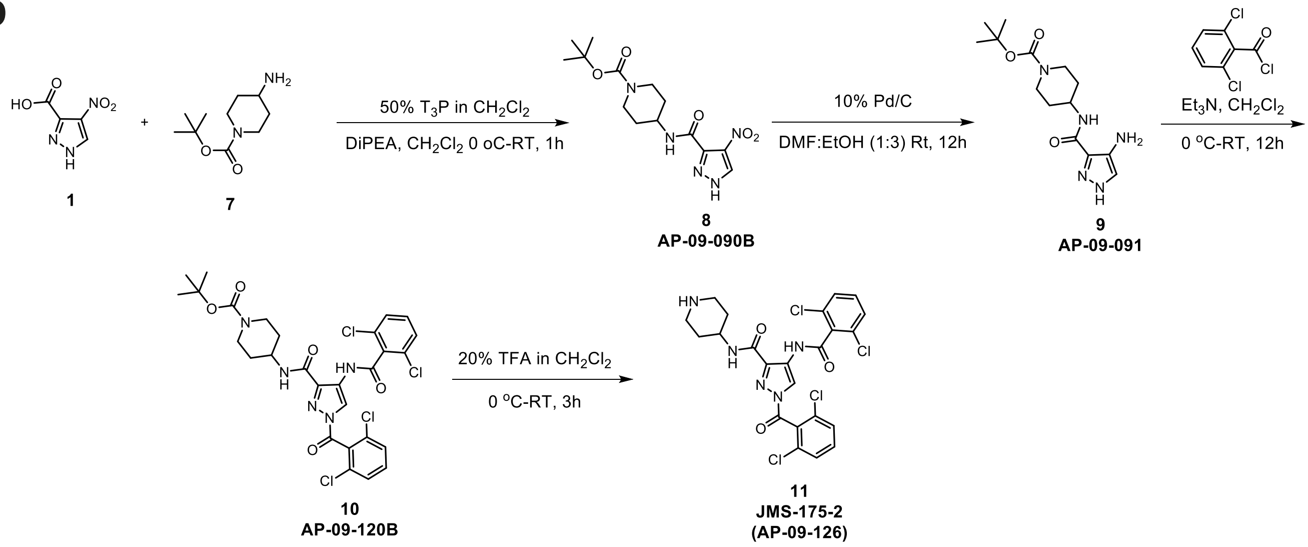

Extended Data Figure 10. Chemical synthesis of BRISC inhibitors
a, Synthesis of FX-171-C (AP-09-131) and **b,** JMS-175-2 (AP-09-126)
