# Supplementary material for: Molecular glues that inhibit specific Zn2+-dependent DUB activity and inflammation
Source: bioRxiv. 2024 Sep 7:2024.09.07.611787. Preprint. [Version 1] doi: 10.1101/2024.09.07.611787 (PMC11398498; doi:10.1101/2024.09.07.611787)
Supplement: Supplementary file 2 [file media-2.pdf]

Extended Data Table 1 - Cryo-EM data collection, refinement, and validation statistics

	**BRISCΔNΔC + FX-171-C**<br>EMD-17980, PDB 8PVY	**BRISCΔNΔC + JMS-175-2**<br>EMD-18009, PDB 8PY2	**BRISC(FL)**
**Data collection and processing**			
Microscope		FEI Titan Krios	
Detector	Thermo Scientific Falcon 4	Thermo Scientific Falcon 4	Thermo Scientific Falcon III
Energy filter	Thermo Scientific SelectrisX		
Energy filter slit (eV)	10		
Magnification	165,000 x	96,000 x	75,000 x
Voltage (kV)	300	300	300
Spot size	7	6	5
Illuminated area (μm)	0.8	1	1.4
Pixel size (Å)	0.71	0.82	1.065
Defocus range (μm)	−1.6 to −2.5	−1.7 to −3.1	−1.7 to −3.1
Total electron dose (e^-/Å^2)	34.97	39.84	88
Exposure (s)	3.43	5.99	1.5
Number of frames	19	36	59
Electron dose per frame (e^-/Å^2)	0.8	0.99	1.49
Movies collected	16,750	7,768	1,610
Acquisition mode	Counting	Counting	Integrating
Symmetry imposed	C1	C1	C1
Initial particle images (no.)	2,458,785	1,616,457	282,442
Final particle images (no.)	632,988	371,872	50,883 (monomer)<br>3,244 (dimer)
Map resolution (Å)	3.02	3.32	8.1 (monomer)<br>25 (dimer)
FSC threshold	0.143	0.143	0.143
Map resolution range (Å)	2.8 - 5.4	3.1 - 7.8	
**Refinement**			
Initial models used (PDB code)	6R8F, 6H3C	6R8F, 6H3C	
Model resolution (Å)	2.7	3.1	
FSC threshold	0.143	0.143	
Model composition			
Non-hydrogen atoms	35797	35797	
Protein residues	4436	4436	
Ligands	ZN: 4<br>FX-171-C: 2	ZN: 4<br>JMS-175-2: 2	
B factors (Å)			
Protein	123.43	101.11	
Ligands	32.47	49.28	
R.M.S.D. from ideal geometry			
Bond lengths (Å)	0.006	0.005	
Bond angles (°)	1.232	0.993	
Validation			
MolProbity score	1.77	1.71	
Clashscore	5.05	4.9	
Ramachandran plot			
Favoured (%)	91.53	92.83	
Allowed (%)	8.31	7.08	
Disallowed (%)	0.16	0.09	
Rotamer outliers (%)	0.3	0.45	
Cβ outliers (%)	0.05	0	
Cis proline (%)	0	0	
CaBLAM outliers (%)	4.79	4.39	

Extended Data Table 2 - HDX-MS Data Summary Table

Data Set	BRISC + DMSO	BRISC + FX-171-C
HDX reaction details	50 mM potassium phosphate, 200 mM NaCl, pD 7.5, 95 % D2O, 4°C	50 mM potassium phosphate, 200 mM NaCl, pD 7.5, 95 % D2O, 4°C
HDX time course (min)	0, 0.5, 1, 10, 60	
HDX control samples	Maximally-labelled controls were not performed.	
Back exchange	Not applicable	
Number of peptides	BRCC36: 110<br>Abraxas2ΔC: 71<br>BRCC45: 110<br>MERIT40ΔN: 89	BRCC36: 111<br>Abraxas2ΔC: 71<br>BRCC45: 109<br>MERIT40ΔN: 89
Sequence coverage (%)	BRCC36: 94.30<br>Abraxas2ΔC: 86.89<br>BRCC45: 88.37<br>MERIT40ΔN: 92.66	BRCC36: 94.30<br>Abraxas2ΔC: 86.89<br>BRCC45: 88.37<br>MERIT40ΔN: 92.66
Average peptide length / Redundancy	BRCC36: 9.87/3.64<br>Abraxas2ΔC: 11.76/3.60<br>BRCC45: 9.99/3.21<br>MERIT40ΔN: 9.21/3.42	BRCC36: 9.88/3.68<br>Abraxas2ΔC: 11.76/3.60<br>BRCC45: 10.00/3.19<br>MERIT40ΔN: 9.21/3.42
Replicates (biological or technical)	3 technical	3 technical
Repeatability (average SD)	BRCC36: 0.1185<br>Abraxas2ΔC: 0.1186<br>BRCC45: 0.0861<br>MERIT40ΔN: 0.0927	BRCC36: 0.0578<br>Abraxas2ΔC: 0.0623<br>BRCC45: 0.0620<br>MERIT40ΔN: 0.0584
Significant differences in HDX (delta HDX > X D)	Reference	99% CI in summed data
